# Nonuniform STM Contrast of Self-Assembled Tri-*n*-octyl-triazatriangulenium Tetrafluoroborate on HOPG

**DOI:** 10.1021/acsomega.3c06454

**Published:** 2023-10-03

**Authors:** Sergii Snegir, Yannick J. Dappe, Dmytro Sysoiev, Thomas Huhn, Elke Scheer

**Affiliations:** † Department of Physics, University of Konstanz, Universitätsstraße 10, Konstanz 78464, Germany; ‡ SPEC, CEA, CNRS, Université Paris-Saclay, CEA Saclay, Gif-sur-Yvette Cédex 91191, France; § Department of Chemistry, University of Konstanz, Universitätsstraße 10, Konstanz 78464, Germany

## Abstract

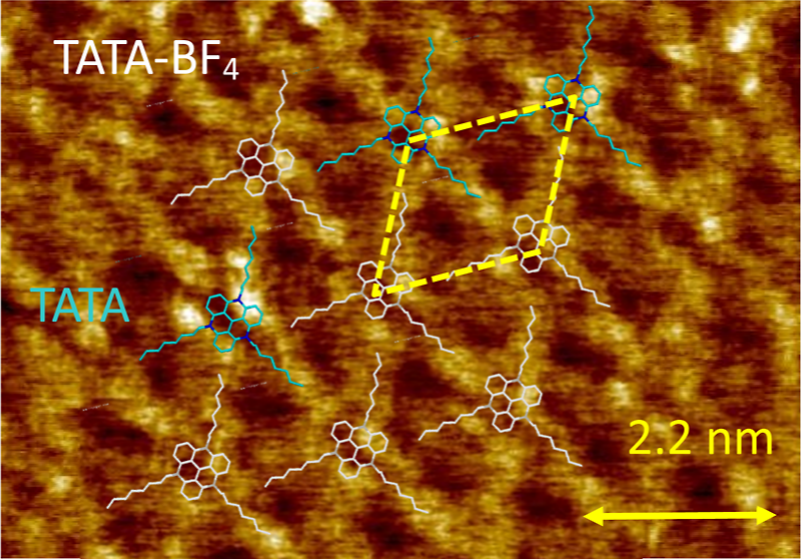

We have assembled 4,8,12-tri-*n*-octyl-4,8,12-triazatrianguleniumtetrafluoroborate (TATA-BF_4_) on highly oriented pyrolytic graphite (HOPG) and have studied the structure and tunneling properties of this self-assembled monolayer (SAM) using scanning tunneling microscopy (STM) under ambient conditions. We show that the triazatriangulenium cations TATA^+^ form hexagonally packed structures driven by the interaction between the aromatic core and the HOPG lattice, as evidenced by density functional theory (DFT) modeling. According to the DFT results, the three alkyl chains of the platform tend to follow the main crystallographic directions of HOPG, leading to a different STM appearance. The STM contrast of the SAM shows that the monolayer is formed by two types of species, namely, TATA^+^ with BF_4_^–^ counterions on top and without them. The cationic TATA^+^ platform gives rise to a seemingly higher appearance than neutral TATA-BF_4_, in contrast to observations made on metallic substrates. The variation of the STM tunneling parameters does not change the relative difference of contrast, revealing the stability of both species on HOPG. DFT calculations show that TATA-BF_4_ on HOPG has sufficient binding energy to resist dissociation into TATA^+^ and BF_4_^–^, which might occur under the action of the electric field in the tunneling gap during STM scanning.

## Introduction

Triazatriangulenium (TATA) salts make up a class of very stable fluorescent dyes with a cationic organic core. TATA^+^ cations are classically synthesized by nucleophilic displacement of all methoxy groups of tris-2,6-dimethoxyphenyl methanol. This is done using a primary *n*-alkyl amine, followed by elimination of water by aid of an acid.^1^ Depending on the nature of the acid, TATAs with different counterions are accessible.^2^ Upon reaction with suitable nucleophiles, uncharged TATA species with the nucleophile covalently bound to the central carbon atom are accessible.^3^ Inspired by this idea, the TATA^+^ core has been functionalized with different azobenzene derivatives, and the platform was found to self-organize on Au surfaces.^3,4^ The high symmetry and π-conjugated planar structure of cationic as well as covalently bound TATAs allowed the formation of extended two-dimensional (2D) supramolecular assemblies on the surface.^4−9^ Moreover, by variation of the peripheral *n*-alkyl chain length, the platform surface density and thus the distance of the attached functional units have been adjusted.^10^

Despite the significant progress of using TATA^+^ as a platform, the unique properties of the cationic core, stabilized by different counterions, have not yet been well explored. Some limited number of studies showed that combining cationic TATA^+^ salts with anion receptors leads to columnar self-assembly of molecules in solution and thus to the formation of organogels consisting of submicrometer-scale fibers.^2^ In these structures, counterions (BF_4_^**–**^, Cl^–^, or Br^–^) play the role of a binding agent, forming the cationic planar TATA^+^ aromatic core with a receptor anion. After deposition of TATA-BF_4_ on a free-electron rich Au surface, the counterions are obscured.^10^ Neither X-ray photoelectron spectroscopy (XPS) nor electrochemical STM in a HClO_4_ electrolyte could detect BF_4_^**–**^ moieties on the surface.^11^ Instead, the authors attribute this behavior to a strong interaction of the monolayer with the Au(111) substrate, leading to a screening of the positive charge of the cation and removal of the corresponding anion after adsorption of the monolayer.^5,11,12^ Recently, we succeeded in clarifying the whereabouts of the BF_4_^**–**^ anion on Au(111) by heat-aided deposition of TATA-BF_4_ from 1,2,4-trichlorobenzene solvent.^13^ The counterions could be localized only under specific tunneling conditions. We detected two types of molecular packing on the Au surface and showed that 15–20% of the TATA^+^ entities within a self-assembled monolayer (SAM) still contained a BF_4_^**–**^ counterion on their top. Some BF_4_ species were found directly on Au(111) trapped in the empty meshes of the SAM. Additionally, density functional theory (DFT) calculations showed that the loss of BF_4_^**–**^ counterions can be explained by the decrease of the strength of the ionic bond between the TATA^+^ core and their counterions due to the interaction of the π-system with the Au lattice.^13^

In the current study, we make efforts to self-assemble TATA-BF_4_ from a 1,2,4-trichlorobenzene solution on a heated HOPG substrate. HOPG was used in this study to test our hypothesis that Au would be particularly efficient in promoting the dissociation of TATA-BF_4_ during the assembly of the monolayer. We assumed that HOPG would interact less strongly with the π-system of the TATA^+^ core, whereas the interaction of the *n*-alkyl chains with the underlying HOPG lattice would dominate due to the known epitaxy of *n*-alkanes with the graphite lattice.^14−17^ Under these conditions, we assumed that the number of self-assembled undissociated TATA-BF_4_ entities would be significantly higher compared to that on Au(111).

## Experimental Section

Synthesis and purification of TATA–BF_4_ with *n*-octyl side chains (R = –[CH_2_]_7_–CH_3_) were performed following [1]. The corresponding NMR spectroscopy studies are presented in our recent paper.^13^ The powder was stored at −18 °C under a N_2_ atmosphere. All STM measurements were performed with freshly prepared solutions of TATA-BF_4_ in 1,2,4-trichlorobenzene. We have used the same concentration of 1.3 × 10^–7^ mL^–1^ as in our previous study, which avoided the formation of a second molecular layer.^5,12^

### STM Measurements

All SAMs were studied under ambient atmospheric conditions using a commercial STM equipped with a low-current head (Bruker, Nanoscope 3A). The calibration of the STM piezoceramics was controlled in advance using a freshly cleaved HOPG surface. The STM tip was prepared by mechanical cutting of a Pt/Ir (80:20) wire. For each SAM, several STM images were recorded in the constant-current mode with the current set point ranging from 10 to 25 pA and tip bias from 0.1 to 0.4 V. Images were obtained for two different samples using four different tips to check the reproducibility and to ensure that the results are free from artifacts. All molecule–molecule distances given in the paper are averaged from different images and along different crystallographic orientations to minimize errors caused by the thermal drift of the STM tip when working under ambient conditions. Postfiltering of the noise in the obtained images was done with the commercial software package SPIP (Digital Surf).

The solution of TATA-BF_4_ was deposited on freshly cleaved HOPG substrates (Micro to Nano, The Netherlands). The substrate with the deposited solution was left on a heated plate (∼50 °C) under ambient atmospheric pressure in a fume cabinet before the STM measurements. After about 1 h of drying, a visual inspection of the substrate revealed complete evaporation of the solvent, and the STM images showed the SAM formation.

The statistical analysis of STM images for defining the ratio of TATA^+^ cores to TATA-BF_4_ within a monitored SAM was done based on the relative height difference.

### Computational Methods

The experimental results are complemented by periodic DFT calculations performed with the localized orbital code Fireball.^18^ This approach uses a self-consistent version of the Harris-Foulkes local density approximation (LDA) functional,^19,20^ instead of the traditional Kohn–Sham functional based on the electronic density. As a result, the total charge leading to the electrostatic potential of the system is approximated as the superposition of spherical charges around each atom. In the Fireball simulation package, self-consistency is achieved over the occupation numbers through the Harris functional.^21^ We built the optimized basis set using a linear combination of wave functions, one from the atom in its ground state and the other one from the atom in its first excited state. This combination allows us to smoothen the decay of the radial part of the wave function and optimize the overlaps between two neighboring atoms.^22^ The LDA exchange–correlation energy is calculated using the efficient multicenter-weighted exchange–correlation density approximation (McWEDA).^23,24^ In the present work, the cutoff radii for the wave functions’ radial part (for s, sp, and spd basis sets) defining the optimized basis set are the following (in atomic units): *r*_s_ = 4.1 for the H atom; *r*_s_ = 4.5, 4.2, and 4.1; *r*_p_ = 4.5, 4.2, and 4.1 for the C, N, and F atoms, respectively; *r*_s_ = 4.5; *r*_p_ = 4.9; and *r*_d_ = 5.2 for the B atom. These optimized basis sets have been well tested in previous works on molecular self-assembly on metallic surfaces.^13,25,26^ Periodic calculations have been performed using a set of 8 × 8 × 1 *k*-points in the surface Brillouin zone of graphene. Finally, we have used a well-established perturbation theory approach combined with the DFT formalism in order to take into account a weak repulsive Coulombic and attractive van der Waals interaction for structural optimization of the molecule adsorption on a single layer of graphene or in the TATA network.^27−29^ Because of van der Waals interactions between the graphene layers, the distance between the TATA and the second layer would be around 6 Å, resulting in only a very weak interaction with no significant influence on the adsorption energy.

Using this approach, we determined the adsorption energy of the TATA^+^ ion on graphene. The adsorption energy is defined in a standard manner as the difference between the total energy of the system and the energy of the isolated ion and the isolated graphene surface, both calculated independently. To this end, we have considered a 14 × 14-graphene unit cell with the TATA^+^ core on top. The core and its arms were fully optimized before setting them on the graphene surface. Then, the whole structure has been optimized until the forces went below 0.1 eV·Å^–1^. The interaction energy of the molecule with the surface was calculated as a function of the molecule–surface distance, taking into account weak repulsive and van der Waals interactions. From the obtained equilibrium structure, we have determined the projected density of states (PDOS) of the different species involved in this study in comparison with their PDOS when adsorbed on a gold surface. Finally, we determined the two-dimensional network of TATA platforms.

## Results and Discussion

### Experimental Observations

**Figure 1:**
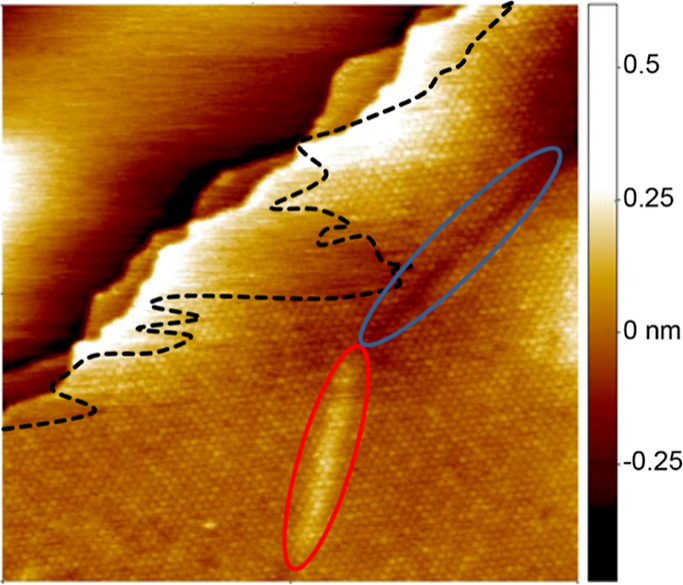
STM image (132.8 × 132.8 nm^2^) of a SAM of TATA-BF_4_ on HOPG (STM parameters: *U*_t_ = 0.25 V and *I*_t_ = 12 pA). The black dashed curve highlights the border between the areas with and without the SAM. The red and blue ellipses are explained in the text. The height color code is used for all STM images throughout the study.

Figure 1 represents a 66.4 × 66.4 nm^2^ area of the HOPG surface with two large terraces. The top left terrace with a smooth STM contrast has no indication of a molecular assembly, whereas the right bottom terrace is almost completely covered by tiny spots forming a periodic pattern. Each of these spots corresponds to the position of a single TATA (see below). The black dashed curve designates the border between the SAM and the disordered region. The observed SAM in Figure 1 forms a single domain since it has rotational symmetry observed at 120°. The observed domain contains several STM contrast peculiarities that are different in nature. Two elongated broad lines with a bright (red markup) and dark (blue markup) STM contrast highlight the surface area where the STM tunneling conditions are not uniform due to a lattice mismatch of the underlying graphite sheets. This stems from either a structural defect or a slight misorientation of a lower lying HOPG layer with respect to the top layer. The resultant wave functions in this area adopt either a maximum or a minimum, leading to the formation of a bright or dark STM contrast, respectively. Despite this effect, the structure of the SAM in these regions remains uniform.

**Figure 2:**
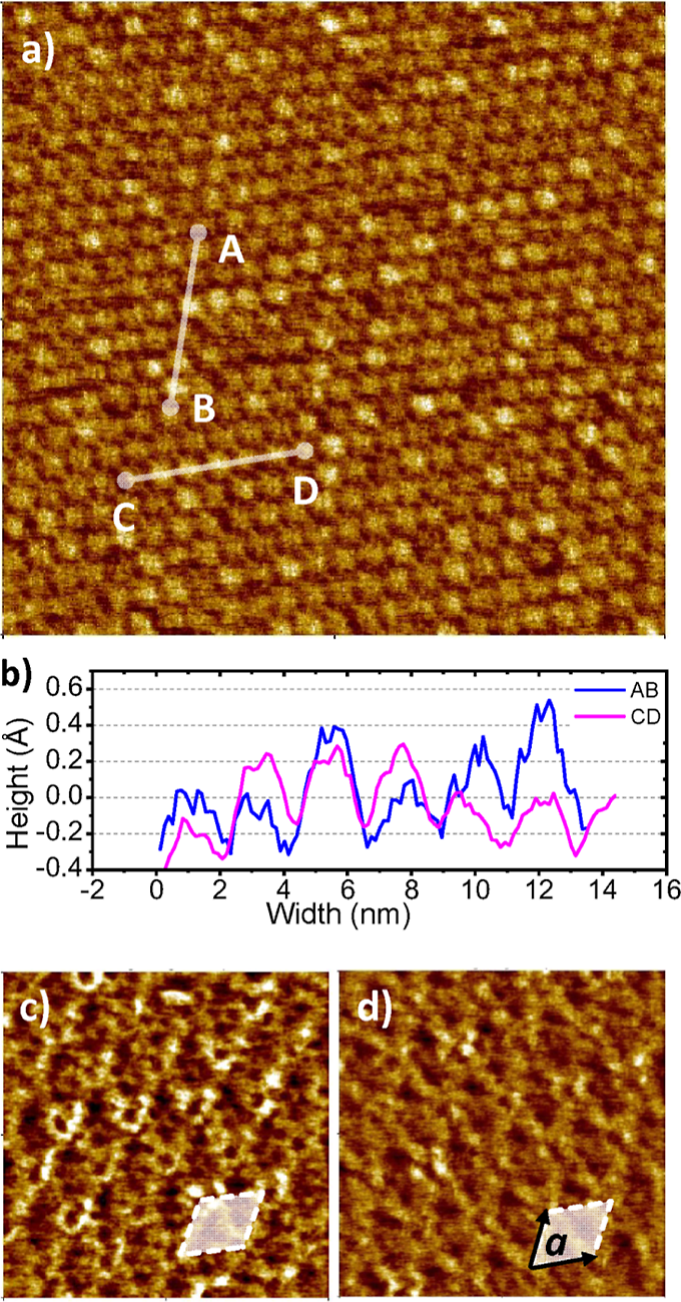
(a) STM image (50.5 × 50.5 nm^2^) of a SAM of TATA-BF_4_ deposited on HOPG. The corresponding STM parameters are *U*_t_ = 0.25 V and *I*_t_ = 12 pA. (b) Cross sections along the corresponding directions in (a). (c,d) STM images (11.3 × 11.3 nm^2^) of an area of (a) recorded under *U*_t_ = 0.3 V, *I*_t_ = 15 pA and *U*_t_ = 0.3 V, *I*_t_ = 14 pA, respectively. The white shaded diamonds in (c,d) indicate the hexagonal unit cell with lattice parameter *a*. The height color code is the same as that in Figure 1.

The analysis of the STM contrast of Figure 1 revealed that entities within a SAM may have a different STM appearance. Figure 2a shows an enlarged area of Figure 1 free from distinct defects of the HOPG layers. The image shows a part of a SAM with irregular STM contrast. The cross sections A–B and C–D along the arbitrarily selected rows of several neighboring bright spots show ∼0.4 Å larger relative height compared to others (Figure 2b), revealing higher local electron tunneling probability. It was counted that among 529 entities imaged in Figure 2a, 128 are showing a relative height between 0.2 and 0.4 Å, suggesting that about 24% of arbitrarily located species show a brighter contrast. We explain the slight variation of the relative height of TATA^+^ (brightness of spots in Figure 2) by disorder in the HOPG substrate. This disorder leads to a small variation of the interaction between the two topmost graphene layers of HOPG and hence a variation of the effective charge of TATA^+^, which is crucial for the STM contrast as we explain in the Discussion section. Scanning the same surface area using different STM tunneling parameters did not change the positions of the bright species. However, upon scanning, local distortions in the vicinity of these species became apparent under certain tunneling parameters.

By variation of the tunneling parameters, different molecular orbitals are probed. The resultant STM contrast of the species underwent a significant change from a large uniform spot (*U*_t_ = 0.25 V and *I*_t_ = 12 pA, Figure 2a) to clearly distinguishable ring-like structures (*U*_t_ = 0.3 V and *I*_t_ = 15 pA, Figure 2c) and finally to a combination of interconnected elongated objects (*U*_t_ = 0.3 V and *I*_t_ = 14 pA, Figure 2d). Cross-sectional analysis of all the observed 2D structures in Figure 2 under the above STM tunneling conditions allowed for reliable identification of a hexagonal arrangement of TATAs within a SAM with a lattice constant of *a* = 2.2 ± 0.1 nm. The minor variation of the current between Figure 2c,d reflects the shape of some molecular orbitals (MOs) which contribute to the current. Although several MOs can be energetically close to each other, their shapes may differ. By a slight decrease in the tunneling current from 0.15 to 0.14 pA, the distance between the STM tip and the sample is increased. This alters the overlap between the wave functions of the metal tip and the MOs of the sample.

### Theoretical Modeling

**Figure 3:**
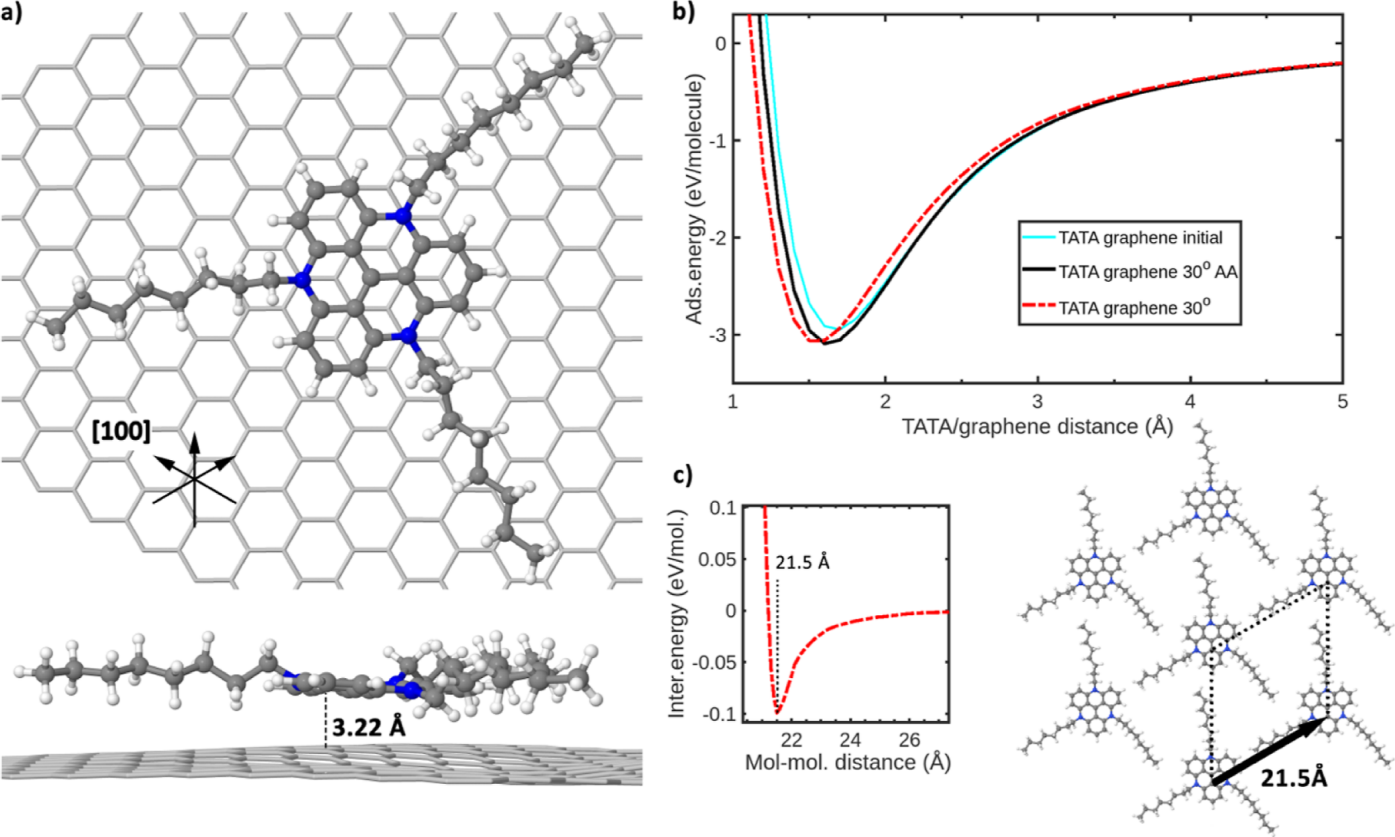
(a) Top and side view of the DFT-optimized configuration of the TATA^+^ ion adsorbed on graphene. The color code is dark gray for carbon, blue for nitrogen, white for hydrogen, and light gray for carbon atoms in graphene. (b) Evolution of the adsorption energy for different adsorption configurations of TATA^+^ on graphene. (c) Evolution of the molecule–molecule interaction energy in the hexagonal molecular network as a function of the intermolecular distance.

In order to determine the adsorption energy of TATA on HOPG, we placed the TATA^+^ ion in different positions on a graphene plane, considering the alignment of one alkyl chain along the [110] direction.^14−17^ The graphene plane was used as the model of the top layer of HOPG. As such, we initially have considered a configuration of the TATA^+^ core being rotated with respect to the graphene hexagonal rings by 30°, then a rotation of TATA^+^ by additional 30° to align the core with the hexagonal rings of the graphene layer, and finally a translation to give an AB-like stacking with respect to the graphene layer. The latter one turns out to be the most favorable position and is represented in Figure 3a. This configuration has been calculated after full DFT optimization and taking into account van der Waals interactions, as described above. The corresponding adsorption energy curves as a function of the molecule–graphene distance are represented in Figure 3b. The obtained energy lies around 3 eV per TATA^+^ ion for an equilibrium distance of 1.6 Å between the lowest hydrogen atom of the ion and the graphene plane, *i.e.* 3.32 Å, between the aromatic core of the ion and the graphene plane (indicated in the bottom of Figure 3a). This corresponds to the usual equilibrium distance between graphitic materials.^30^ In order to characterize the experimentally observed TATA molecular network on HOPG and to compare the molecule–graphene and molecule–molecule interactions, we have also theoretically modeled the potential network by constructing a hexagonal unit cell of 7 TATA^+^ ions, as represented in Figure 3c. We have then calculated the interaction energy between the central ion and its next neighbors in the cell, for different intermolecular distances, using the previous formalism. As a result, we find an equilibrium distance of 21.5 Å between two neighboring ions, corresponding to the van der Waals equilibrium distance. This is in good agreement with the experimental observations. The corresponding interaction energy variation is represented in Figure 3c.

### Discussion

**Figure 4:**
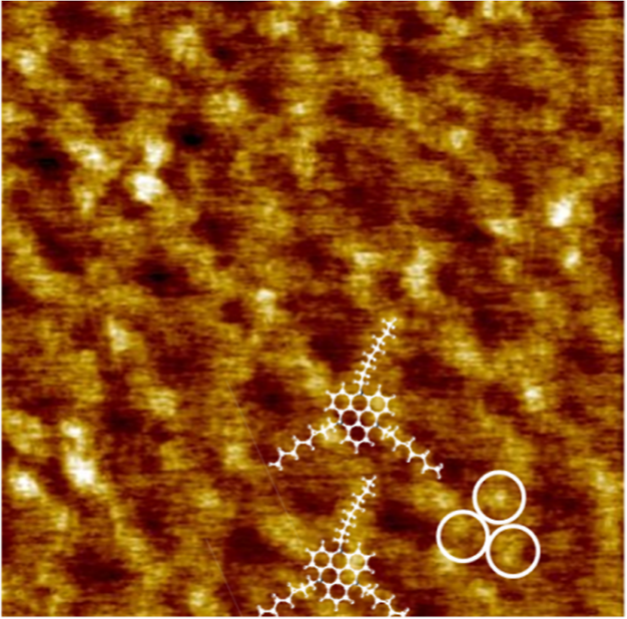
STM image (8.9 × 8.9 nm^2^) with superimposed DFT-optimized configuration of TATA. The STM parameters are *U*_t_ = 0.3 V and *I*_t_ = 14 pA.

Figure 4 shows a small part (8.9 × 8.9 nm^2^) of Figure 2d with the superimposed chemical structure of *n*-octyl-TATA^+^. According to DFT calculations, the structure of the molecule has a distorted star-like geometry because it allows gaining a minimum of energy on graphene (Figure 3a). BF_4_^**–**^ was intentionally removed from the structure to simplify the figure. The aromatic cores of the ion structures were placed directly above the surface areas that under the monitored tunneling conditions appeared as ring-like objects (Figure 2c,d). A similar ring-like contrast was observed for TATA on Au(111), reflecting positions of the molecular orbital of the central cores of the TATA entities.^13^ However, the STM contrast of the molecular core on HOPG has peculiarities that are observed under *U*_t_ = 0.3 V and *I*_t_ = 14 pA. We attribute these peculiarities to a strong epitaxy of the aromatic TATA^+^ core with the underlying HOPG lattice (Figure 3a). Our DFT calculations strongly favor an orientation of the six hexagonal rings of the TATA^+^ core with respect to the graphene lattice. This is similar to the case of two adjacent graphene planes in a consecutive AB pattern by translation along one C–C bond (see above).^14−16^ Hence, the carbon atoms of the TATA^+^ core are located in an alternating fashion above the center of a graphene hexagon and above the carbon atom of the underlying graphene. The latter coorientation creates specific tunneling conditions, allowing STM visualization of overlapping atoms of TATA^+^ and graphene (Figure 3a). Three of these atoms in the TATA^+^ core are nitrogen atoms, which might lead to the observed variation in STM contrast compared with the situation with two overlapping carbon atoms. Therefore, this effect can explain the presence of the observed bright spots in the STM contrast (white rings in Figure 4). By application of this hypothesis, the exact position of the aromatic core of TATA^+^ on the STM image (Figure 4) can be explained as well as the exact positions of the alkyl chains. The alkyl chains lie right above the elongated 2D objects in the recorded STM image (Figure 4). At first sight, it seems counterintuitive that the alkyl chains that are known to be “invisible” for STM on the Au(111) surface have distinguishable STM contrast on HOPG. However, it is also known that alkanes tend to orient themselves along [110] crystallographic directions and hence tend to form ideal epitaxial structures on a HOPG lattice.^14−17^ Such epitaxy leads to the appearance of periodically repeated protrusions due to created resonant tunneling conditions, allowing visualization of the entire length of the alkyl chains.^14−16^ This situation holds true only for two of the three alkyl chains, whereas the third chain remains invisible due to a slight misfit with the HOPG lattice. From DFT calculations (Figure 3a), it becomes apparent that two alkyl chains at least partially lie above the carbon atoms along [110], while the third alkyl chain cannot accommodate a position along or even close to the [110] direction. Apparently, the interaction of the aromatic core of TATA^+^ with graphene outweighs the simultaneous epitaxy of all the three alkyl chains on graphene. From these results, we deduce that the adsorption configuration of TATA^+^ on HOPG is imposed mainly by the aromatic core of TATA^+^, whereas the alkyl chains are occupying favorable positions on the lattice by tilting their N–C–C angle and/or by bending the chain (see the DFT-optimized structure in Figure 3a).

At the same time, the packing density of the SAM is defined exclusively by the length of the alkyl chains. TATA platforms cannot be packed any closer because of the steric hindrance between neighboring entities (Figure 3c). This leads to a minimum intermolecular interaction energy of 0.1 eV/mol at a separation distance of 21.5 Å ≈ 9 × *T*_HOPG_ (*T*_HOPG_ = 2.46 Å). The analysis of STM images recorded under different tunneling conditions (given in Figure 2a–d) shows that the same self-assembly mechanism is active for both species with normal and brighter STM contrast.

One open question so far is whether the bright species [with ∼0.4 Å bigger relative height for the tunneling conditions of Figure 1 compared to “dark” molecules] correspond to TATAs that still bear their BF_4_^–^ counterions on top or to naked TATA^+^ ions instead.

**Figure 5:**
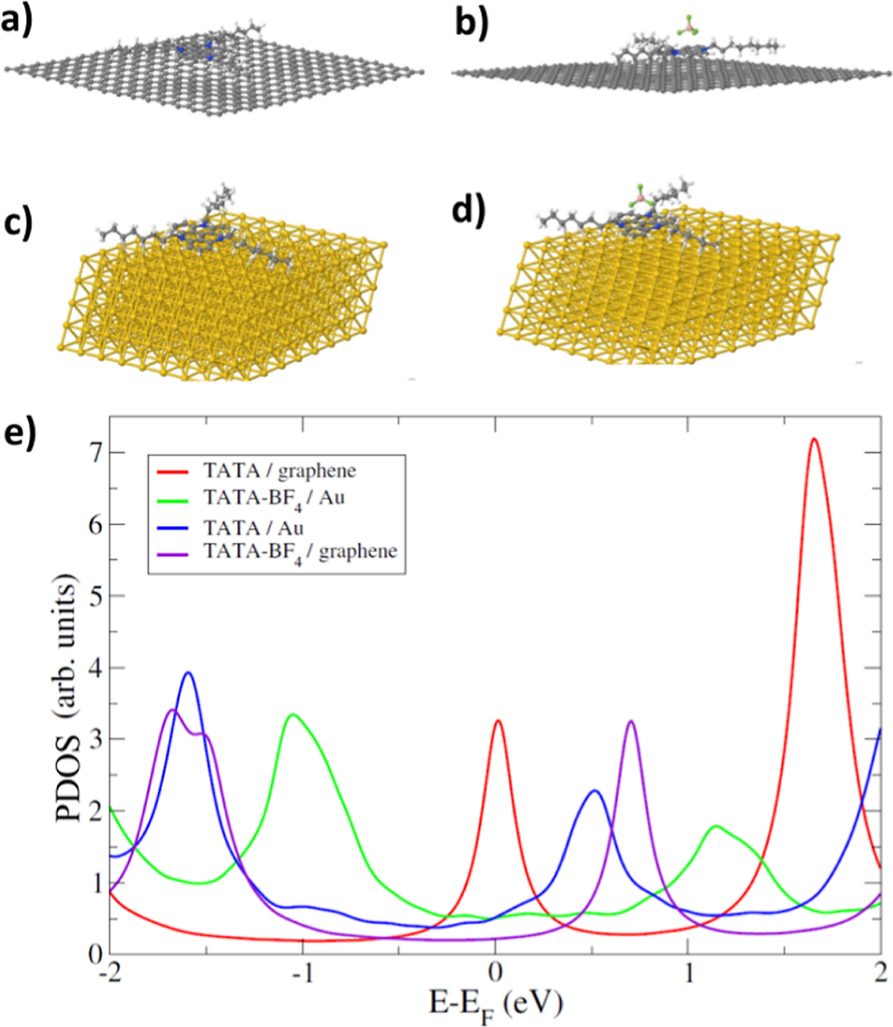
Geometrical configuration of (a) TATA^+^, (b) TATA-BF_4_ on graphene, (c) TATA^+^ on Au(111), and (d) TATA-BF_4_ on Au(111). (e) Calculated PDOS for the four configurations shown in panels (a–d).

To address this question, we calculated the DOS for TATA^+^ and TATA-BF_4_ on graphene in the configuration shown in Figure 5a,b respectively. The DOS for the TATA^+^ ion shows a pronounced resonance at the Fermi level *E*_F_ corresponding to the highest occupied molecular orbital (HOMO) of the ion. It is located close to *E*_F_ because of the weaker van der Waals interaction of TATA^+^ with the substrate, which prevents the ion from being fully neutralized from graphene. In contrast, the HOMO of the neutral molecule TATA-BF_4_ is located more than 1 eV below *E*_F_. We argue that the enhanced DOS at *E*_F_ of TATA^+^ may result in an enhanced electronic transmission probability to the STM tip and, hence, in a brighter STM appearance. The absolute value of the transmission critically depends on the position of the molecule on the substrate as well as the distance between the TATA^+^ and the BF_4_^–^ counterion and the distance to the STM tip. These parameters are not known in the experiment, hindering us from performing meaningful transmission calculations.

We hence posit that the bright protrusions in the STM figure do NOT show the undissociated TATA-BF_4_ molecules but instead the naked TATA^+^ ions. Counting these latter species, we find a fraction of roughly 24% bright molecules, meaning that 76% of the molecules stay undissociated. This interpretation is additionally supported by the slightly varying STM appearance of the bright spots when applying larger voltages. As can be seen in Figure 5e, the full width at half-maximum (fwhm) of the resonance at *E*_F_ of TATA^+^ is about 0.4 eV. Increasing the tunneling voltage, *U*_t_, from 0.25 to 0.3 V slightly changed the appearance of the bright species, while the dark species remain unaffected. In our earlier study of TATA-BF_4_ on Au(111),^13^ we observed the opposite effect in both aspects: undissociated TATA-BF_4_ appeared as bright spots and with a relative abundance of 15–20%. An important difference between the two systems is that the bright spots reflect different aspects of HOPG and Au(111). While on Au(111) they can be seen as topographic contrast (higher elevation due to the presence of the counterions), they signal electronic effects in the case of HOPG. For completeness, we show in Figure 5e also the DOS for TATA^+^ and TATA-BF_4_ on Au (111), where in both cases, the HOMO is well below *E*_F_ and broadened, indicative of the partial charge transfer due to the strong interaction with the metal substrate.

As mentioned in the Introduction section, in our earlier study of TATA-BF_4_ on Au(111), we were able to observe the split-off BF_4_^**–**^ counterions in the meshes of the TATA-SAMs.^13^ On HOPG, we do not detect free counterions, most likely because of their smaller amount and weak interaction with HOPG, which might enhance their mobility. Moreover, in the current study, all bright spots stay constant during several STM scans and during application of different tunneling bias and currents, which was not the case for TATA-BF_4_ on a Au surface. Such observations support the idea that the bright species correspond to the naked TATA^+^ platforms.

Since about a quarter of all species lose their counterions, one may ask at which moment in the sample preparation they are cleaved off the platform. One possibility would be that part of the molecules predissociate in the solution and can adsorb on HOPG in both predissociated (TATA^+^) and undissociated (TATA-BF_4_) forms. The final formation of the SAM leads to the stabilization of either TATA-BF_4_ or TATA^+^ species on HOPG. Obviously, more integral methods such as XPS might bring additional insights to answer the question. However, the self-assembly of TATA-BF_4_ does not lead to the formation of sufficiently large domains for a distinct XPS study. Also, particular approaches applied to promote the SAM formation were insufficient to detect BF_4_^**–**^ on the surface by XPS.^11^

## Conclusions

A SAM of TATA-BF_4_ on HOPG is formed by mainly van der Waals interaction between the aromatic core of TATA^+^ and the substrate. The 6-fold rotational symmetry of graphene (top layer of HOPG) partially imposes the alkyl chains to orient with respect to the graphene lattice, forming a hexagonal molecular packing, revealed by a star-like structure of the entities on the surface. The presence of BF_4_^**–**^ counterions on the majority of the TATA^+^ platforms does not impair the substrate–molecule interaction and therefore does not distort the hexagonal packing of the SAM. Undissociated TATA-BF_4_ can be easily detected on HOPG using different STM tunneling conditions. The main reason for this behavior is that the electric field in the tunneling gap is insufficient to overcome the dissociation energy of TATA-BF_4_.

## Supporting Information Available

Details on theoretical modeling of TATA^+^ and TATA-BF_4_ on HOPG including the result for different stacking geometries, information on noise filtering of some of the STM images, details of the calculation of the projected density of states, and structural data of the adsorbed molecules (PDF)
