# Supplementary material for: Nonuniform STM Contrast of Self-Assembled Tri-n-octyl-triazatriangulenium Tetrafluoroborate on HOPG
Source: ACS Omega. 2023 Oct 3;8(41):38766–72. doi: 10.1021/acsomega.3c06454 (PMC10586247; doi:10.1021/acsomega.3c06454)
Supplement: Supplementary file 1 — ao3c06454_si_001.pdf [file ao3c06454_si_001.pdf]

Supplementary information

Non-Uniform STM Contrast of Self-Assembled tri-n-octyl-triazatriangulenium tetrafluoroborate on HOPG

Sergii Snegir [a], Yannick J. Dappe [b], Dmytro Sysoiev [c], Thomas Huhn [c] and Elke Scheer [*a]

a. Department of Physics, University of Konstanz, Universitätsstr. 10, 78464 Konstanz, Germany

b. SPEC, CEA, CNRS, Université Paris-Saclay, CEA Saclay, 91191 Gif-sur-Yvette Cédex, France.

c. Department of Chemistry, University of Konstanz, Universitätsstr. 10, 78464 Konstanz, Germany.

* corresponding author

1. Theoretical modeling of TATA+ and TATA-BF_4 on HOPG

To determine the adsorption energy of $TATA^+$ cations on HOPG, we placed this ion in different positions on a graphene plane constructed from 14 x 14 unit cells, considering the alignment of one alkyl chain along the [110] direction, as observed experimentally. The graphene plane was used as the model of the top layer of HOPG. As such, we initially have considered a configuration of the $TATA^+$ core being rotated with respect to the graphene hexagonal rings by 30° (Figure S1). Next, a configuration with the $TATA^+$ core being aligned with the hexagons of the graphene leading to a stacking in an AA fashion (Figure S2) was investigated. Finally, a configuration with the $TATA^+$ core being translated by half the unit cell to give an AB-like stacking with regard to the graphene (Figure S3) turned out to be the most favorable position. This configuration has been calculated after full DFT optimization and taking into account van der Waals interactions as described above.

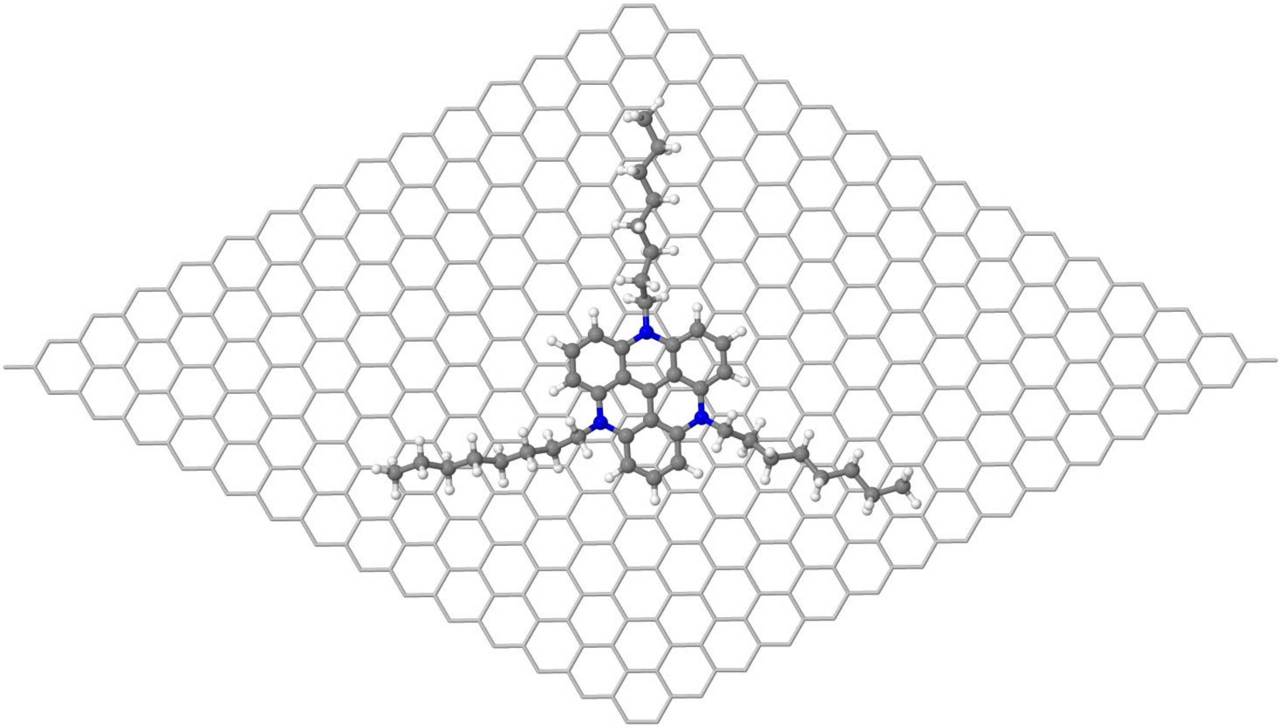

Figure S1: Top view of DFT-optimized configuration of a $TATA^+$ cation randomly adsorbed on a 14 x 14 unit cell graphene sheet.

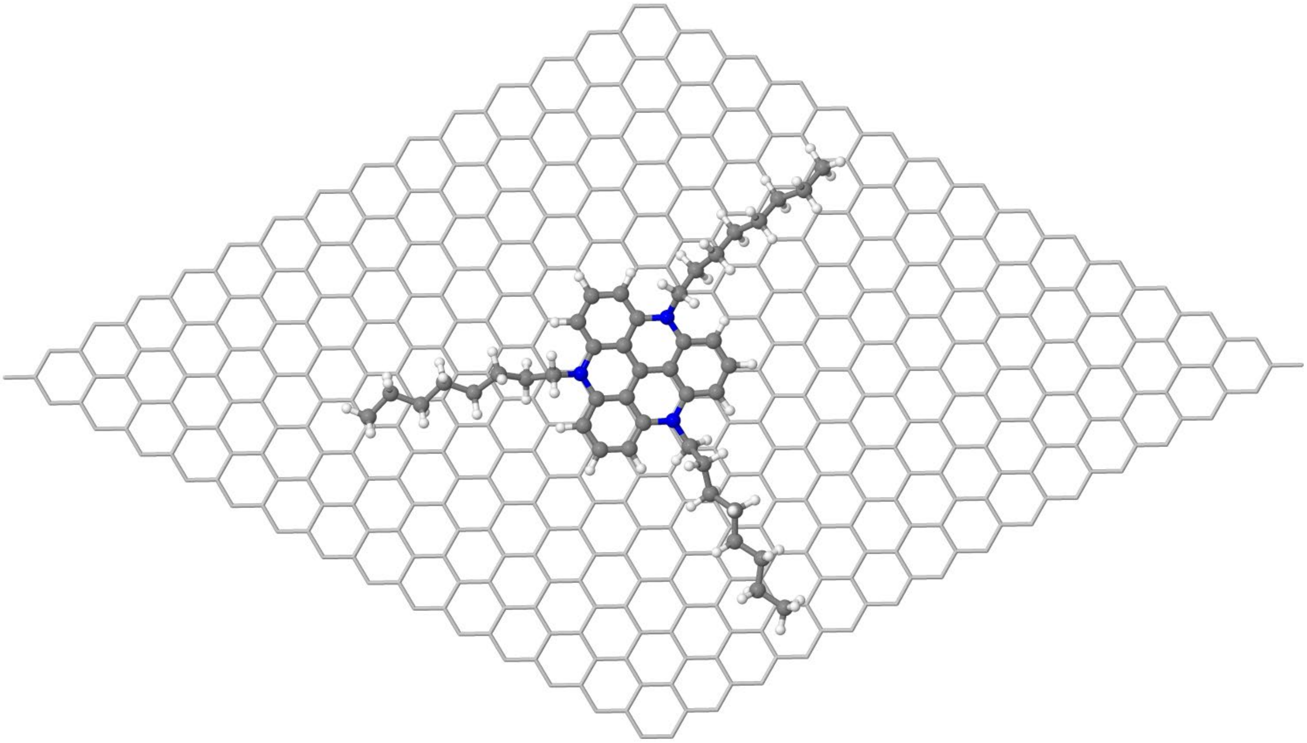

Figure S2: Top view of DFT-optimized configuration of a $TATA^+$ cation adsorbed on a 14 x 14 unit cell graphene sheet in an AA-like stacking with respect to the graphene sheet.

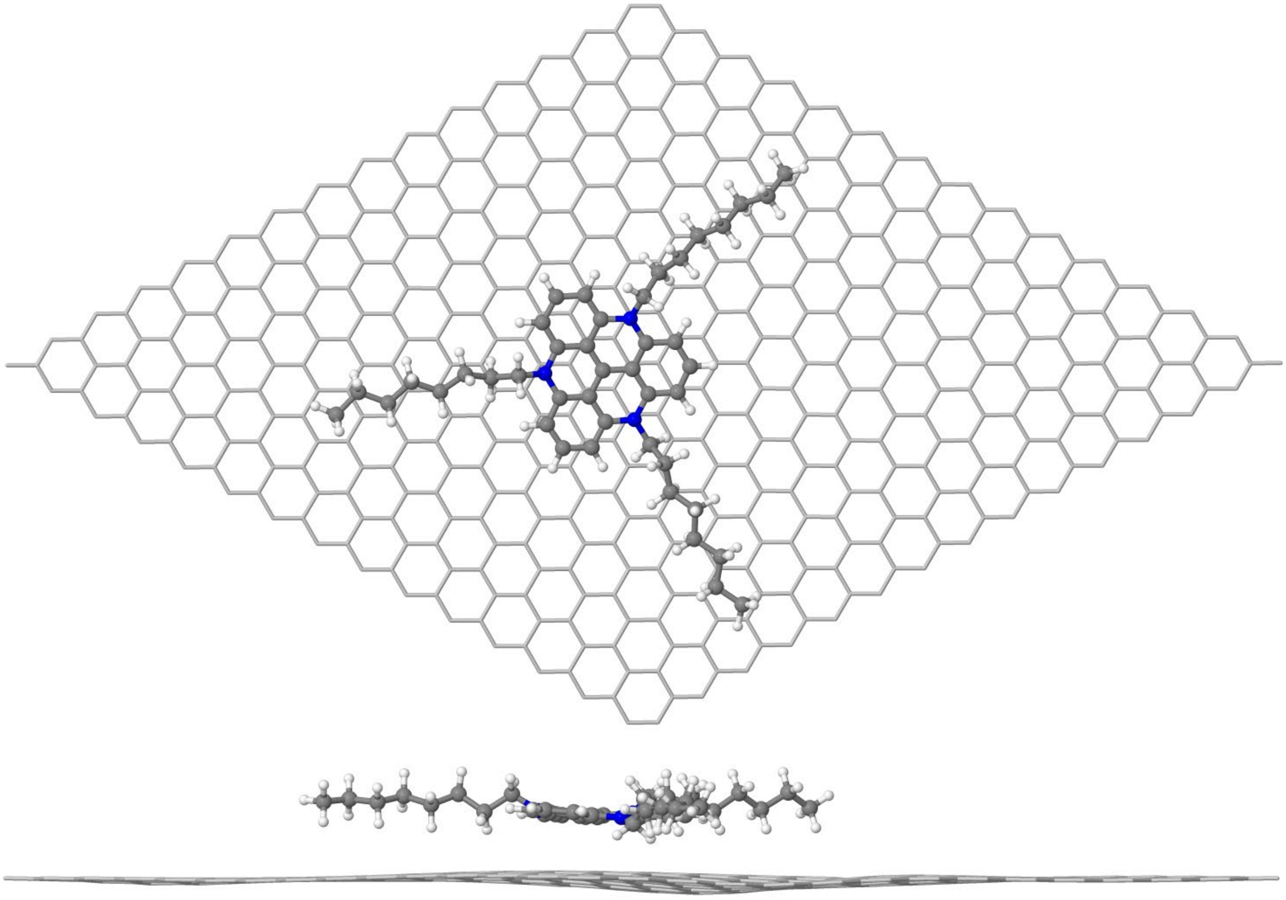

Figure S3: Top (Bottom): Top (side) view of DFT-optimized configuration of a $TATA^+$ cation adsorbed on a 14 x 14 unit cell graphene sheet in an AB-like stacking with respect to the graphene sheet. (original Figure 3a) of the manuscript).

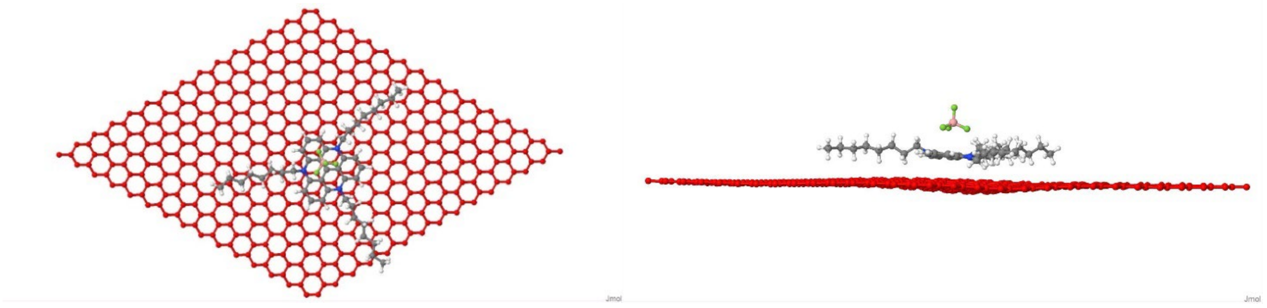

Figure S4. Top and side view of DFT-optimized configuration of a TATA-BF_4 molecule adsorbed on graphene. Chemical elements are color-coded (gray for carbon, blue for nitrogen, white for hydrogen, pink for boron, green for fluorine and red for carbon atoms in graphene).

2. Filtering of the noise in some STM images

The quality of all images is limited by the thermal drift and external acoustical noise, since all measurements have to be done in ambient atmospheric environment to gain a thermally induced self-organization of the molecules. This is the typical situation in ambient STM, and the figure quality is state–of-the-art for this method. As an example for the noise level in ambient STM measurements Fig. S5 shows the original data of Fig. 4 of the manuscript before (left) and after noise filtering (right) using the well-established software package SPIP (Digital Surf)[1].

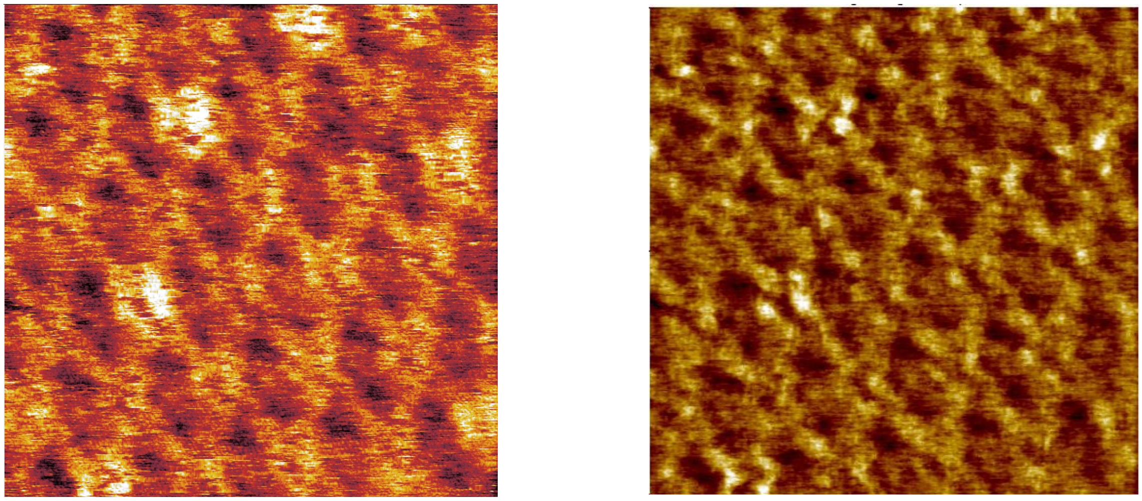

a) b)

Figure S5 Original (a) and filtered (b) image of the data presented in Fig. 4 in the main manuscript using the well-established software package SPIP [1]

3. Calculations of the Projected Density of States (PDOS)

In order to fully discriminate the species observed in the STM images, we have performed electronic structure calculations on four different potential configurations on graphene and Au(111), respectively. Namely, we have considered $TATA^+$/graphene and $TATA^+$ BF_4^-/graphene and the same on Au (111). The atomic structure of these configurations is represented in Figure 5a) to d) in the main text. As for the other calculations, structural optimizations and electronic structure calculations have been performed using the Fireball code[1]. The whole methodology has been fully detailed previously.[2,3] The corresponding PDOS for the four configurations are represented in Figure 5 e) in the main text. As a result, we observe a pronounced maximum of the PDOS close to E_F for $TATA^+$/graphene, while no maximum is close to E_F for the other three configurations. Comparing with TATA-BF_4/graphene, we observe a similar width of the resonances while on Au(111) they are broadened. These trends can

be explained with the much weaker van der Waals interaction between the TATA species and graphene compared to Au(111). The position of a molecular resonance close to E_F, as observed for $TATA^+$/graphene, signals the ionic nature of the $TATA^+$ platform and the weak charge transfer between $TATA^+$ and graphene. A high DOS at E_F is a mandatory ingredient of a high electronic transmission.[2,3] Although the absolute position of the resonance will depend on the positioning of the molecule with respect to the graphene lattice, we can safely expect a higher electronic transmission for the $TATA^+$ than for the neutral molecule TATA-BF_4 provided a similar distance of the STM tip is chosen and similar molecular orbitals and electronic wavefunctions of the substrate are involved. We refrain from performing explicit transport calculations since the exact shape and position of the STM tip is not known in our experiment and will have a dominating influence on the absolute value of the transmission. Hence, these calculations give us the trend that when measuring the TATA with and without the BF_4^- ion, there will be a clear difference in the obtained STM contrast.

4. Structural data of the adsorbed molecules

We provide here the .xyz files for the molecular configurations (TATA-BF4 on graphene and TATA+ on graphene, all coordinates are in Å).

Molecule/graphene

561

F	4.347126	4.431258	-3.750866
B	4.384186	4.527560	-5.284053
H	3.256508	10.491077	-6.596839
H	9.585537	0.840273	-6.908947
H	6.772925	1.353502	-7.078246
H	3.112756	15.915123	-6.926646
H	2.596051	8.016015	-6.929848
H	3.361588	13.282127	-6.675926
H	7.692868	2.853195	-7.205642
H	4.350078	8.136688	-7.083899
F	3.209674	5.424278	-5.800775
H	11.112085	-1.364608	-6.712569
H	8.339820	-0.365068	-7.230600
F	5.732798	5.153029	-5.767500
H	-3.069712	-0.401113	-6.715137
H	12.511357	-3.581504	-6.618170
F	4.250881	3.100333	-5.910325

H	-5.261515	-2.070202	-7.033566
H	-0.935374	1.673110	-7.034173
H	9.787222	-2.372133	-7.290007
C	7.218174	2.058061	-7.797176
C	9.072212	0.296780	-7.712981
C	3.415683	8.083475	-7.660377
H	-7.672466	-3.267604	-7.327651
H	0.759696	16.766874	-7.231304
H	1.925841	17.889587	-7.942020
H	4.536201	10.955788	-7.711872
C	3.476910	10.676746	-7.656105
H	-0.379172	0.085465	-7.578330
H	1.203324	14.237153	-7.369826
H	11.198182	-4.506836	-7.337572
C	10.635371	-1.757646	-7.620264
C	2.609105	15.811047	-7.895464
C	3.108112	13.264759	-7.743853
H	-7.289380	-0.765848	-7.615944
H	1.575864	11.717736	-7.784249
C	1.508467	16.882559	-8.018384
C	12.083266	-3.903016	-7.576156
H	-1.142652	6.408386	-8.335580
H	4.034787	13.479636	-8.291955
H	13.406299	-5.640529	-7.732640
C	2.061010	14.369238	-8.041538
C	-3.093471	-0.464760	-7.810354
N	3.439391	6.796193	-8.438032
C	2.600389	11.858677	-8.151330
C	0.980224	6.679667	-8.415620
C	-0.874314	1.008126	-7.904164
H	5.880048	-0.047460	-8.580281
C	-0.175049	5.909826	-8.419596
H	-2.620959	-1.416857	-8.085465
H	-5.072227	0.415461	-7.831302
N	6.074091	2.668974	-8.559136
H	0.894803	7.760223	-8.345176
C	2.240232	6.058783	-8.526631
H	5.885126	7.993494	-8.591568
C	4.935766	0.487071	-8.621959
C	3.245061	9.382789	-8.493282
C	5.896520	6.907090	-8.618012
H	3.372095	15.981936	-8.666321
C	4.695429	6.172391	-8.616999

C	10.093365	-0.583560	-8.475401
H	3.802612	-1.331004	-8.633117
C	7.117260	6.242406	-8.662164
H	-1.084892	3.979290	-8.469956
C	7.205971	4.854214	-8.669965
C	-0.146536	4.522807	-8.517602
C	4.898884	1.894630	-8.666669
C	6.033352	4.076870	-8.675295
H	8.184784	4.380649	-8.679774
H	8.037442	6.829936	-8.680274
C	3.753293	-0.240606	-8.656761
C	8.301749	1.293483	-8.616998
C	-5.392919	-1.721721	-8.064930
C	-4.565185	-0.433771	-8.307349
C	4.757897	4.740256	-8.735420
C	2.294270	4.629694	-8.690224
C	3.558353	3.974728	-8.753262
C	-6.901186	-1.489205	-8.344435
C	1.082451	3.852427	-8.672116
C	2.506013	0.370482	-8.709925
C	3.623532	2.549643	-8.768979
H	1.617410	-0.252941	-8.698458
C	-2.301452	0.702994	-8.449849
C	2.412068	1.774346	-8.761909
C	-7.749514	-2.773910	-8.298739
C	11.641431	-2.655732	-8.381662
H	-8.803933	-2.553508	-8.483743
N	1.171476	2.445474	-8.777775
H	-2.898964	1.620429	-8.379808
H	-5.017819	-2.520420	-8.719050
H	2.238991	9.405341	-8.928567
H	10.926675	0.035341	-8.831816
C	13.108286	-4.773394	-8.326933
C	-0.067549	1.666643	-9.065203
H	8.996204	1.996764	-9.093184
H	12.525859	-2.064694	-8.652184
H	14.009678	-4.202373	-8.563495
H	-7.421619	-3.481748	-9.064501
H	3.939203	9.374258	-9.340183
H	0.996484	16.808625	-8.981189
H	9.611461	-0.994985	-9.371471
H	7.828699	0.734749	-9.433161
H	2.537394	11.824313	-9.244930

H	1.677524	14.238138	-9.061490
H	-0.744004	2.332841	-9.614263
H	-4.567514	-0.223221	-9.384869
H	11.189071	-2.982911	-9.327072
H	0.193895	0.874419	-9.778537
H	-2.221698	0.482918	-9.522324
H	-7.011892	-1.024949	-9.332457
H	12.695801	-5.140497	-9.270941
C	5.586687	10.675136	-11.610440
C	8.060643	9.240450	-11.505603
C	8.057147	10.670029	-11.410902
C	6.822371	11.385530	-11.469993
C	5.590746	9.246818	-11.718219
C	4.350554	11.389560	-11.647473
C	6.826915	8.531869	-11.677271
C	9.295729	8.524842	-11.448450
C	3.111456	10.677478	-11.734006
C	3.113382	9.247906	-11.818589
C	4.352881	8.535536	-11.838289
C	9.294996	11.382816	-11.299964
C	10.532827	9.236280	-11.317868
C	1.872559	11.388231	-11.683199
C	4.347576	12.819348	-11.574485
C	9.300170	7.096682	-11.562894
C	6.819973	12.816536	-11.411939
C	0.633851	10.672523	-11.646731
C	10.531905	10.668265	-11.264678
C	6.832001	7.104850	-11.805609
C	1.872642	8.532941	-11.825808
C	0.636676	9.242072	-11.710782
C	5.585021	13.531482	-11.474779
C	10.534740	6.380357	-11.500512
C	1.870559	12.819496	-11.630932
C	8.067864	6.389474	-11.752700
C	3.111034	13.533173	-11.601093
C	11.770028	8.522037	-11.292222
C	4.355389	7.106684	-11.938313
C	11.771484	7.090921	-11.363323
C	9.294578	12.815502	-11.277794
C	-0.601170	11.383287	-11.538327
C	8.058476	13.530085	-11.327223
C	-0.600737	8.525463	-11.651009
C	5.594462	6.394386	-11.945703

C	10.538965	4.952186	-11.610432
C	1.875020	7.103705	-11.917508
C	-1.834693	9.235089	-11.508600
C	-1.837920	10.667023	-11.461872
C	0.633024	13.530834	-11.581193
C	8.071513	4.960462	-11.860167
C	-0.604704	12.815212	-11.524229
C	3.113677	6.393585	-11.997839
C	11.773233	11.383357	-11.232155
C	9.305347	4.244163	-11.786482
C	13.008906	6.376839	-11.344654
C	-0.599999	7.096781	-11.757336
C	11.773914	4.236258	-11.554695
C	5.583100	14.962874	-11.450603
C	13.011329	9.236736	-11.248861
C	3.108723	14.963757	-11.569242
C	13.010977	4.946713	-11.424000
C	-3.072919	8.518912	-11.451089
C	0.633987	6.389039	-11.909560
C	5.596748	4.964373	-12.037429
C	6.834534	4.249808	-12.001697
C	10.535766	13.530545	-11.259665
C	8.057997	14.962605	-11.334736
C	13.011177	10.669556	-11.240616
C	-3.074689	11.380375	-11.381967
C	-3.071578	7.088651	-11.544757
C	-1.838920	6.380502	-11.709889
C	11.778237	2.807697	-11.657591
C	9.308670	2.814583	-11.878230
C	4.347715	15.677000	-11.510603
C	0.630827	14.962593	-11.574056
C	11.773210	12.816377	-11.249461
C	-1.841177	13.528365	-11.461996
C	6.822213	15.676704	-11.391911
C	14.250067	7.091090	-11.296191
C	10.543546	2.098293	-11.810861
C	1.871099	15.676809	-11.585922
C	-4.309841	9.232092	-11.356962
C	3.115481	4.963647	-12.091006
C	14.249498	8.523349	-11.269740
C	6.834936	2.817697	-12.053150
C	4.354914	4.250769	-12.115056
C	-3.079179	12.813298	-11.396364

C	-4.313947	10.665271	-11.335025
C	8.071025	2.103275	-12.002089
C	14.248621	4.232827	-11.412443
C	-4.311748	6.374080	-11.513240
C	13.013823	2.092206	-11.611242
C	0.634549	4.960860	-12.028390
C	-1.840273	4.954111	-11.847023
C	10.546381	0.667844	-11.880321
C	10.535637	14.962815	-11.299984
C	14.251201	2.803124	-11.493118
C	9.298606	15.677048	-11.326067
C	-5.551076	8.518068	-11.341841
C	5.593477	2.102834	-12.114925
C	-0.606204	15.675247	-11.540756
C	-1.844680	14.960370	-11.485290
C	8.071000	0.669658	-12.017765
C	-5.548431	7.086395	-11.406072
C	-4.312419	4.946575	-11.637459
C	5.587242	-2.185921	-11.856444
C	6.828978	-0.045009	-12.025977
C	6.828112	-1.475347	-11.932890
C	9.307135	-0.045142	-11.964676
C	5.587923	-3.612543	-11.713330
C	1.872716	4.249667	-12.122942
C	4.355512	2.817375	-12.152092
C	-3.080308	4.238873	-11.805990
C	15.488078	6.378185	-11.323139
C	14.252167	11.384590	-11.287897
C	5.592621	0.670006	-12.079299
C	13.017874	0.663135	-11.700850
C	11.781421	-0.047986	-11.822042
C	4.345868	17.108405	-11.510766
C	15.489171	4.946485	-11.361374
C	3.110550	-3.609653	-11.690356
C	4.347625	-4.322813	-11.623827
C	4.350550	-1.473161	-11.930761
C	8.064299	-2.190382	-11.893064
C	-0.607430	4.246382	-12.014321
C	13.013908	13.530911	-11.301333
C	-5.552999	11.380474	-11.324436
C	3.109611	-2.184481	-11.856139
C	9.305708	-1.478231	-11.935036
C	1.869395	17.107991	-11.579329

C	1.869920	-4.322735	-11.628564
C	-4.317129	13.527795	-11.380290
C	6.824753	-4.328490	-11.688101
C	6.821453	17.108330	-11.418552
C	-5.553947	4.233249	-11.620995
C	4.350856	-0.044803	-12.061855
C	15.490379	9.238416	-11.307437
C	8.064452	-3.619620	-11.788714
C	0.633197	-3.612516	-11.726039
C	-6.790487	6.374149	-11.418825
C	-6.790241	9.233567	-11.326754
C	4.348944	-5.753199	-11.515660
C	-3.082923	2.811354	-11.931684
C	-5.556206	2.806237	-11.742377
C	-6.789669	4.944536	-11.510080
C	10.542502	-2.193170	-11.899958
C	-4.322939	2.096397	-11.882951
C	0.632084	-2.187377	-11.889608
C	11.783159	-1.479541	-11.863616
C	15.488629	2.089478	-11.486217
C	1.871008	-5.752885	-11.515794
C	-5.557067	12.813214	-11.354992
C	-0.607040	-4.327458	-11.680718
C	14.251140	12.816728	-11.328566
C	1.872891	-1.474407	-11.956960
C	3.109353	17.821751	-11.554314
C	-6.793880	10.666593	-11.329504
C	15.489274	10.670803	-11.329890
C	14.253724	-0.052073	-11.659883
C	5.585325	17.822011	-11.473294
C	3.113445	2.102133	-12.182480
C	-1.844052	-3.618456	-11.774864
C	-1.845964	-2.191537	-11.909661
C	-3.081816	15.674365	-11.464868
C	-0.608412	17.106730	-11.556997
C	6.826011	-5.758496	-11.589232
C	-4.324130	0.665757	-11.952180
C	3.108319	-6.467540	-11.470930
C	-3.084540	-1.480767	-11.958958
C	3.113998	0.668480	-12.134066
C	11.776030	15.676908	-11.347751
C	9.300627	-4.335965	-11.782575
C	-0.605632	-1.478002	-11.980058

C	-3.087538	-0.048913	-12.021259
C	9.298214	17.108520	-11.377391
C	5.585872	-6.469914	-11.508266
C	-4.320884	14.959946	-11.422589
C	16.728874	7.093043	-11.350717
C	-6.797378	2.092629	-11.724524
C	1.873676	2.817051	-12.189283
C	-5.564413	-0.049569	-11.900509
C	16.727074	4.233781	-11.390253
C	10.541502	-3.625196	-11.850492
C	0.631399	17.821125	-11.579397
C	-0.606058	-5.757034	-11.570508
C	-4.324331	-2.194824	-11.898933
C	15.491404	0.659597	-11.559774
C	-8.029076	7.088990	-11.389782
C	0.630796	-6.469271	-11.502115
C	13.012947	14.962576	-11.357290
C	-8.031707	8.521023	-11.359826
C	-1.849698	2.100682	-12.077051
C	-3.084140	-4.333775	-11.738384
C	8.061623	17.822365	-11.412500
C	-8.031786	4.232764	-11.529189
C	16.727546	8.525043	-11.356969
C	1.872354	-0.047416	-12.105606
C	-6.799428	0.663331	-11.810071
C	13.018743	-2.194848	-11.815701
C	16.728697	2.802767	-11.434331
C	-5.562706	-1.482130	-11.902523
C	-1.847545	0.666196	-12.095253
C	-0.607715	2.816676	-12.124534
C	-4.321769	-3.624206	-11.808539
C	-8.032419	2.804011	-11.621349
C	14.256824	-1.482253	-11.726500
C	8.062050	-6.475822	-11.605241
C	-0.608302	-0.049710	-12.109362
C	9.301550	-5.765828	-11.697053
C	-8.032023	11.381688	-11.376241
C	-6.795055	13.527911	-11.392687
C	3.109414	-7.901574	-11.434734
C	-1.845918	-6.474145	-11.569164
C	-3.084683	17.106226	-11.503326
C	-3.082757	-5.763336	-11.641923
C	-9.270852	6.377320	-11.431936

C	-9.269500	4.946498	-11.481730
C	-1.845483	17.820452	-11.540854
C	-8.039812	-0.050988	-11.776075
C	-6.802419	-2.196560	-11.851801
C	11.778505	-4.340322	-11.831746
C	5.586817	-7.903261	-11.466944
C	0.631508	2.102217	-12.199641
C	-9.269996	9.236344	-11.399120
C	17.967871	4.948319	-11.404004
C	0.633969	0.665928	-12.169711
C	17.966288	6.379977	-11.398944
C	13.019587	-3.626803	-11.826785
C	-5.558537	15.674489	-11.442158
C	-8.035206	12.813968	-11.408731
C	-9.274014	2.091209	-11.621353
C	3.107286	19.252864	-11.554533
C	0.632011	-7.902961	-11.463852
C	-5.561734	-4.339192	-11.770546
C	16.728499	-0.054153	-11.548617
C	16.728552	11.384772	-11.412754
C	5.583943	19.253156	-11.498675
C	15.490220	13.530514	-11.415516
C	-9.272725	10.668477	-11.413385
C	-8.039830	-1.482840	-11.808541
C	11.775244	17.108371	-11.407970
C	10.538537	17.822546	-11.413260
C	-9.274922	0.661421	-11.688617
C	-6.798304	14.960181	-11.434988
C	4.346275	-8.617849	-11.440595
C	-6.799826	-3.627665	-11.801868
C	17.966508	2.089876	-11.456394
C	0.629807	19.252510	-11.579999
C	15.492572	-2.197002	-11.684801
C	1.869068	-8.617842	-11.442669
C	-10.511123	4.234601	-11.510144
C	-10.508944	7.092115	-11.449329
C	8.063179	-7.907891	-11.550362
C	10.537716	-6.482529	-11.707442
C	17.967308	9.239193	-11.427841
C	-10.510616	2.804074	-11.560329
C	-10.510870	8.523947	-11.445771
C	-4.322464	-6.479822	-11.635987
C	6.823071	-8.620465	-11.493882

C	11.778235	-5.771696	-11.773376
C	14.252334	15.676500	-11.436268
C	17.968347	0.659059	-11.497721
C	-1.844667	-7.906591	-11.524975
C	8.060729	19.253590	-11.461107
C	16.730899	-1.484471	-11.604303
C	-5.559902	-5.768897	-11.691534
C	4.347152	19.966500	-11.534331
C	-4.321754	17.820543	-11.507493
C	16.726983	12.816491	-11.455496
C	1.869964	19.966157	-11.573680
C	9.303725	-18.646300	-11.469572
C	8.066229	-20.791956	-11.477850
C	9.297328	29.977998	-11.749826
C	-5.561430	17.106397	-11.485005
C	8.061100	32.122580	-11.659990
C	-0.607544	-8.619911	-11.488594
C	19.205321	4.235394	-11.442992
C	17.965649	10.670925	-11.458602
C	-11.748891	4.948580	-11.500730
C	-11.750162	6.379844	-11.486596
C	14.255576	-4.341620	-11.778693
C	-1.847361	19.251902	-11.564885
C	19.206331	7.094014	-11.450910
C	19.207001	2.803907	-11.455265
C	6.824054	19.967086	-11.493432
C	6.824449	34.266255	-11.562730
C	10.536428	27.833215	-11.748242
C	15.488978	14.962400	-11.465545
C	6.827751	-22.936737	-11.496549
C	8.063520	-19.359777	-11.502851
C	10.540759	-16.500071	-11.479283
C	8.061596	30.691465	-11.686927
C	15.494620	-3.628255	-11.719484
C	6.824991	32.835512	-11.597813
C	6.825581	-21.505000	-11.514525
C	-0.607339	19.965869	-11.581326
C	19.204558	8.525704	-11.471002
C	9.300730	-17.214018	-11.497641
C	9.299113	28.546603	-11.732822
C	4.347176	-10.051889	-11.463199
C	9.299007	-8.624942	-11.579055
C	10.538998	-7.913359	-11.644463

C	5.585977	36.410774	-11.515709
C	5.587067	34.979686	-11.531438
C	5.588047	-25.081135	-11.511096
C	5.586458	-23.649714	-11.523697
C	1.870269	-10.051685	-11.469274
C	19.205767	-0.054071	-11.508988
C	20.445728	4.949308	-11.474565
C	-4.321132	-7.911247	-11.585008
C	20.443970	6.380686	-11.484017
C	6.826143	-18.645060	-11.554946
C	10.537614	19.253779	-11.468434
C	13.014823	17.822528	-11.468090
C	11.776512	25.690850	-11.666274
C	-3.084061	-8.623515	-11.545894
C	6.824006	-10.053887	-11.495783
C	20.444486	2.090842	-11.483604
C	5.584521	32.121901	-11.575352
C	13.015231	-6.486805	-11.751383
C	6.822202	29.977577	-11.639442
C	5.587666	-20.790732	-11.561591
C	17.967759	-2.198371	-11.584322
C	10.538901	26.402810	-11.690361
C	22.924406	4.949374	-11.510550
C	11.777763	-14.353774	-11.508641
C	22.922975	6.380789	-11.517407
C	10.537961	-15.067693	-11.507856
C	4.346269	34.266354	-11.523426
C	9.300786	19.967650	-11.483315
C	-4.324015	19.252236	-11.545086
C	21.683530	4.235954	-11.497338
C	14.251505	17.108364	-11.484177
C	21.685090	2.804678	-11.498911
C	8.063591	-16.498860	-11.541697
C	5.585353	-19.358670	-11.578857
C	20.446123	0.659706	-11.499978
C	14.256084	-5.773424	-11.757895
C	4.347601	32.835127	-11.537310
C	5.585689	30.690874	-11.591557
C	6.823465	-17.212573	-11.567368
C	21.684879	7.094288	-11.517421
C	4.348271	-22.935935	-11.550392
C	5.583566	-10.768121	-11.489909
C	8.060162	27.832914	-11.672433

C	-3.084297	19.965961	-11.568852
C	3.107113	-10.767107	-11.482901
C	19.207440	-1.485068	-11.541060
C	20.444928	9.239318	-11.525752
C	19.205401	11.384691	-11.529199
C	4.346442	-21.504149	-11.571001
C	21.683215	8.525697	-11.537265
C	4.345545	21.397786	-11.546984
C	6.823596	28.546366	-11.638419
C	-0.606301	-10.053320	-11.503267
C	3.107267	32.121460	-11.523953
C	1.868325	21.397285	-11.575495
C	17.966540	13.530605	-11.532542
C	16.730390	-4.342605	-11.671651
C	20.443243	10.670936	-11.552171
C	9.300340	-10.057332	-11.553095
C	8.060881	-15.066017	-11.548003
C	4.345683	29.977077	-11.566266
C	8.060142	-10.770423	-11.524552
C	4.347435	-18.644102	-11.605023
C	3.108699	30.690361	-11.540360
C	9.300840	-14.352225	-11.533140
C	6.822727	21.398209	-11.526870
C	3.108152	-20.790247	-11.583330
C	5.585925	-16.497225	-11.593247
C	0.630609	-10.767633	-11.503226
C	17.969593	-3.629299	-11.614895
C	11.775356	-8.629428	-11.649873
C	8.062092	26.401750	-11.643071
C	19.203913	12.816737	-11.560341
C	9.299221	25.688604	-11.647870
C	1.868380	29.976537	-11.544480
C	-0.608844	21.397183	-11.594299
C	11.775414	-12.921506	-11.538747
C	11.778100	24.259885	-11.613499
C	5.583924	27.832480	-11.598612
C	4.345443	-17.211358	-11.610325
C	13.015764	-7.917488	-11.692855
C	3.106428	-19.358125	-11.602661
C	13.015313	23.547503	-11.585524
C	16.728387	15.676930	-11.535776
C	3.108482	22.111112	-11.564657
C	4.347063	28.545831	-11.573899

C	13.015266	-12.207938	-11.557459
C	5.584914	-12.201184	-11.525823
C	6.823827	-14.350223	-11.560800
C	5.585606	22.111626	-11.544836
C	5.583656	-15.064337	-11.582649
C	6.821671	-12.917298	-11.540992
C	8.061754	-12.203489	-11.533959
C	9.298481	-12.919542	-11.539049
C	-3.083009	-10.056373	-11.540262
C	0.631256	22.110813	-11.590297
C	11.777389	19.968234	-11.507457
C	3.108297	-12.200110	-11.530633
C	1.868317	-18.643929	-11.600698
C	17.965509	14.962914	-11.562937
C	10.536781	-10.773655	-11.567924
C	13.014111	19.254442	-11.509249
C	15.491935	-6.487513	-11.707415
C	10.538656	-12.205992	-11.546934
C	1.869862	28.545506	-11.563198
C	5.585438	26.401310	-11.590359
C	4.344928	-12.915641	-11.547569
C	6.822521	25.687887	-11.603346
C	9.299651	21.398844	-11.528034
C	-3.085930	21.397451	-11.601956
C	16.731724	-5.773953	-11.675621
C	11.776818	-10.060940	-11.603138
C	4.346584	-14.348703	-11.582782
C	3.107021	27.831993	-11.565493
C	-1.845983	-10.769789	-11.532188
C	8.062700	22.112233	-11.542638
C	3.107898	-16.496462	-11.616762
C	0.629956	27.831929	-11.590396
C	9.301008	24.257718	-11.605171
C	13.013426	-10.776321	-11.595909
C	10.538320	23.544787	-11.592549
C	1.867083	-17.211328	-11.612743
C	-1.845876	22.111001	-11.611998
C	5.584068	23.542774	-11.562512
C	6.824154	24.256765	-11.579922
C	15.491161	17.823440	-11.536340
C	4.345477	25.687443	-11.572633
C	3.106433	-15.063441	-11.603144
C	8.061214	23.543488	-11.576561

C	3.108450	26.400838	-11.570990
C	14.252399	-8.632119	-11.667002
C	3.106919	23.542306	-11.568945
C	4.346963	24.256135	-11.566842
C	14.253622	-10.063198	-11.621325
C	0.631552	-12.200711	-11.544570
C	1.868293	-12.915412	-11.559516
C	13.016342	22.115569	-11.558096
C	10.539614	22.113452	-11.553459
C	16.727868	17.109518	-11.558777
C	15.492652	-7.918855	-11.674618
C	0.629631	-16.497032	-11.600406
C	11.776507	21.400123	-11.542814
C	0.631481	26.400645	-11.601088
C	14.253489	21.402426	-11.548564
C	0.629758	23.542173	-11.597123
C	1.869788	24.255848	-11.582562
C	-0.608232	25.687109	-11.633105
C	1.868310	25.687015	-11.581713
C	1.869256	-14.348400	-11.594021
C	-1.847146	23.542217	-11.637733
C	14.254072	19.969900	-11.539206
C	-0.606951	24.255830	-11.624267
C	0.628922	-15.063904	-11.599220
C	15.490838	19.256271	-11.548492
C	-1.845335	-12.202654	-11.552294
C	-0.608398	-12.916625	-11.564421
C	-0.608010	-14.349668	-11.582176

Ion/graphene

556

H	5.256507	14.291076	-6.450457
H	14.511355	0.218495	-6.471788
H	5.361587	17.082125	-6.529543
H	13.112083	2.435391	-6.566187
H	-1.069712	3.398883	-6.568755
H	11.585535	4.640272	-6.762565
H	5.112752	19.715122	-6.780261
H	4.596050	11.816012	-6.783464
H	-3.261512	1.729795	-6.887184
H	1.064624	5.473105	-6.887791

H	8.772923	5.153501	-6.931864
H	6.350075	11.936685	-6.937515
H	9.692866	6.653194	-7.059260
H	10.339817	3.434931	-7.084215
H	2.759694	20.566873	-7.084920
H	11.787219	1.427864	-7.143625
H	-5.672462	0.532395	-7.181269
H	13.198180	-0.706837	-7.191188
H	3.203322	18.037151	-7.223442
C	14.083265	-0.103016	-7.429774
H	1.620827	3.885463	-7.431948
H	-5.289379	3.034149	-7.469560
C	12.635369	2.042352	-7.473880
C	5.476909	14.476744	-7.509723
C	5.415681	11.883473	-7.513995
H	6.536200	14.755786	-7.565489
C	11.072210	4.096777	-7.566599
H	15.406297	-1.840529	-7.586258
C	5.108111	17.064757	-7.597470
H	3.575863	15.517734	-7.637867
C	9.218170	5.858056	-7.650794
C	-1.093471	3.335236	-7.663972
H	-3.072226	4.215460	-7.684920
C	4.609103	19.611046	-7.749081
C	1.125685	4.808124	-7.757780
H	3.925840	21.689584	-7.795638
C	3.508465	20.682558	-7.872001
C	4.061008	18.169236	-7.895152
C	-3.392917	2.078278	-7.918548
H	-0.620959	2.383141	-7.939079
C	4.600386	15.658675	-8.004946
H	6.034785	17.279634	-8.145571
C	-5.749513	1.026089	-8.152355
C	-2.565183	3.366228	-8.160966
C	15.108285	-0.973395	-8.180550
H	0.857347	10.208384	-8.189197
C	-4.901182	2.310792	-8.198052
H	2.894801	11.560221	-8.198793
H	-0.898964	5.420427	-8.233425
C	13.641429	1.144266	-8.235279
C	2.980220	10.479665	-8.269237
C	1.824950	9.709825	-8.273213
N	5.439389	10.596190	-8.291648

C	-0.301452	4.502993	-8.303466
H	0.915107	7.779289	-8.323572
C	12.093363	3.216438	-8.329017
H	-6.803931	1.246491	-8.337360
C	5.245059	13.182788	-8.346899
C	1.853462	8.322805	-8.371218
C	4.240230	9.858781	-8.380247
N	8.074089	6.468969	-8.412753
H	16.009675	-0.402374	-8.417111
H	7.880047	3.752539	-8.433897
H	7.885124	11.793492	-8.445185
C	6.695426	9.972388	-8.470615
C	10.301747	5.093479	-8.470615
C	7.896518	10.707088	-8.471629
C	6.935765	4.287067	-8.475576
H	5.802609	2.468995	-8.486734
H	14.525857	1.735304	-8.505800
C	5.753292	3.559393	-8.510377
C	9.117257	10.042405	-8.515781
H	5.372092	19.781932	-8.519937
C	6.898882	5.694626	-8.520286
C	9.205968	8.654213	-8.523581
C	3.082450	7.652425	-8.525733
C	8.033350	7.876869	-8.528912
H	10.184782	8.180647	-8.533391
H	10.037440	10.629934	-8.533891
C	4.294266	8.429693	-8.543841
H	3.617408	3.547057	-8.552075
C	4.506011	4.170480	-8.563542
H	-3.017815	1.279578	-8.572665
C	6.757895	8.540252	-8.589036
C	5.558351	7.774727	-8.606878
C	4.412066	5.574344	-8.615524
C	5.623529	6.349642	-8.622595
N	3.171475	6.245472	-8.631392
H	12.926671	3.835340	-8.685432
H	4.238989	13.205339	-8.782184
H	2.996481	20.608623	-8.834806
H	3.677522	18.038136	-8.915105
H	-5.421617	0.318250	-8.918117
C	1.932450	5.466642	-8.918820
H	10.996202	5.796763	-8.946801
H	4.537391	15.624311	-9.098546

H	14.695797	-1.340497	-9.124558
H	13.189068	0.817087	-9.180687
H	-5.011891	2.775049	-9.186073
H	5.939199	13.174255	-9.193799
H	11.611459	2.805013	-9.225087
H	-2.567511	3.576776	-9.238485
H	9.828697	4.534747	-9.286778
H	-0.221699	4.282916	-9.375939
H	1.255994	6.132837	-9.467878
H	2.193893	4.674418	-9.632153
C	11.773232	11.383357	-11.232155
C	13.011176	10.669556	-11.240615
C	13.011328	9.236736	-11.248860
C	11.773210	12.816376	-11.249461
C	10.535766	13.530545	-11.259665
C	10.531905	10.668264	-11.264677
C	14.249498	8.523348	-11.269740
C	9.294577	12.815502	-11.277794
C	14.252167	11.384589	-11.287895
C	11.770027	8.522035	-11.292222
C	14.250066	7.091090	-11.296191
C	9.294995	11.382816	-11.299963
C	10.535636	14.962815	-11.299984
C	13.013908	13.530910	-11.301332
C	15.490379	9.238416	-11.307437
C	10.532826	9.236280	-11.317868
C	15.488077	6.378185	-11.323138
C	-5.552997	11.380473	-11.324436
C	9.298605	15.677047	-11.326067
C	-6.790241	9.233567	-11.326753
C	8.058476	13.530084	-11.327223
C	14.251139	12.816727	-11.328566
C	-6.793878	10.666593	-11.329504
C	15.489274	10.670802	-11.329890
C	8.057997	14.962604	-11.334735
C	-4.313945	10.665271	-11.335024
C	-5.551076	8.518067	-11.341841
C	13.008905	6.376839	-11.344654
C	11.776030	15.676906	-11.347751
C	16.728874	7.093041	-11.350716
C	-5.557067	12.813214	-11.354991
C	-4.309840	9.232091	-11.356961
C	16.727546	8.525043	-11.356968

C	13.012947	14.962576	-11.357290
C	-8.031707	8.521022	-11.359826
C	15.489171	4.946485	-11.361373
C	11.771482	7.090919	-11.363322
C	-8.032023	11.381688	-11.376241
C	9.298213	17.108519	-11.377390
C	-4.317129	13.527794	-11.380290
C	-3.074688	11.380375	-11.381966
C	-8.029075	7.088987	-11.389782
C	16.727074	4.233780	-11.390252
C	6.822212	15.676704	-11.391911
C	-6.795054	13.527910	-11.392687
C	-3.079178	12.813297	-11.396364
C	17.966287	6.379977	-11.398944
C	-9.269996	9.236344	-11.399119
C	17.967870	4.948318	-11.404004
C	-5.548428	7.086395	-11.406072
C	11.775244	17.108371	-11.407970
C	-8.035206	12.813966	-11.408730
C	8.057147	10.670028	-11.410902
C	6.819973	12.816535	-11.411939
C	14.248620	4.232827	-11.412442
C	8.061623	17.822365	-11.412499
C	16.728552	11.384771	-11.412753
C	10.538536	17.822545	-11.413259
C	-9.272724	10.668476	-11.413384
C	15.490220	13.530514	-11.415516
C	6.821453	17.108329	-11.418552
C	-6.790485	6.374149	-11.418825
C	-4.320884	14.959946	-11.422589
C	13.010977	4.946710	-11.423999
C	17.967307	9.239193	-11.427841
C	-9.270851	6.377320	-11.431936
C	16.728697	2.802766	-11.434331
C	3.109414	-7.901574	-11.434734
C	-6.798301	14.960181	-11.434988
C	14.252333	15.676500	-11.436268
C	4.346275	-8.617848	-11.440595
C	-5.558537	15.674488	-11.442156
C	1.869068	-8.617841	-11.442669
C	19.205321	4.235394	-11.442992
C	-10.510870	8.523946	-11.445771
C	9.295728	8.524841	-11.448449

C	-10.508943	7.092113	-11.449329
C	5.583100	14.962873	-11.450601
C	19.206330	7.094013	-11.450910
C	-3.072919	8.518912	-11.451088
C	19.207001	2.803907	-11.455265
C	16.726983	12.816489	-11.455496
C	17.966508	2.089875	-11.456393
C	17.965648	10.670925	-11.458602
C	8.060729	19.253589	-11.461107
C	-1.837920	10.667023	-11.461871
C	-1.841177	13.528365	-11.461994
C	4.347176	-10.051887	-11.463198
C	0.632011	-7.902961	-11.463851
C	-3.081815	15.674364	-11.464866
C	15.488977	14.962400	-11.465545
C	5.586814	-7.903260	-11.466943
C	13.014822	17.822527	-11.468090
C	10.537613	19.253779	-11.468434
C	1.870269	-10.051685	-11.469274
C	9.303725	-18.646300	-11.469571
C	6.822371	11.385529	-11.469993
C	3.108318	-6.467539	-11.470928
C	19.204557	8.525703	-11.471002
C	5.585325	17.822009	-11.473292
C	20.445727	4.949308	-11.474565
C	5.585021	13.531482	-11.474778
C	8.066228	-20.791955	-11.477850
C	10.540758	-16.500070	-11.479283
C	-9.269500	4.946498	-11.481730
C	3.107113	-10.767106	-11.482901
C	9.300786	19.967649	-11.483314
C	20.444486	2.090841	-11.483603
C	20.443970	6.380683	-11.484016
C	14.251504	17.108364	-11.484177
C	-5.561429	17.106397	-11.485004
C	-1.844680	14.960369	-11.485290
C	15.488628	2.089478	-11.486216
C	-11.750161	6.379844	-11.486596
C	-0.607544	-8.619911	-11.488594
C	5.583566	-10.768121	-11.489909
C	14.251201	2.803124	-11.493118
C	6.824054	19.967085	-11.493432
C	6.823070	-8.620464	-11.493881

C	6.824004	-10.053886	-11.495782
C	6.827751	-22.936737	-11.496548
C	21.683530	4.235954	-11.497336
C	9.300729	-17.214016	-11.497640
C	17.968347	0.659059	-11.497721
C	5.583942	19.253156	-11.498675
C	21.685089	2.804678	-11.498910
C	20.446123	0.659706	-11.499978
C	10.534739	6.380357	-11.500512
C	-11.748891	4.948578	-11.500730
C	0.630796	-6.469271	-11.502114
C	8.063520	-19.359777	-11.502850
C	0.630609	-10.767633	-11.503225
C	-0.606301	-10.053319	-11.503266
C	-3.084683	17.106224	-11.503325
C	8.060643	9.240448	-11.505603
C	11.777388	19.968233	-11.507457
C	-4.321754	17.820543	-11.507493
C	10.537960	-15.067693	-11.507856
C	5.585872	-6.469914	-11.508266
C	-1.834692	9.235089	-11.508599
C	11.777763	-14.353773	-11.508641
C	19.205767	-0.054071	-11.508988
C	13.014110	19.254442	-11.509249
C	-6.789669	4.944536	-11.510080
C	-10.511122	4.234600	-11.510144
C	22.924406	4.949372	-11.510550
C	4.347715	15.676999	-11.510602
C	4.345868	17.108405	-11.510766
C	5.588047	-25.081133	-11.511096
C	-4.311747	6.374080	-11.513239
C	6.825580	-21.504999	-11.514525
C	4.348944	-5.753199	-11.515660
C	5.585975	36.410774	-11.515708
C	1.871008	-5.752885	-11.515793
C	22.922975	6.380789	-11.517407
C	21.684878	7.094286	-11.517421
C	4.346269	34.266353	-11.523426
C	5.586458	-23.649712	-11.523697
C	3.107266	32.121459	-11.523953
C	-0.604704	12.815212	-11.524229
C	8.060142	-10.770421	-11.524551
C	-1.844667	-7.906589	-11.524974

C	20.444928	9.239318	-11.525752
C	5.584914	-12.201183	-11.525823
C	6.822727	21.398209	-11.526870
C	9.299651	21.398844	-11.528033
C	-8.031786	4.232764	-11.529189
C	19.205400	11.384691	-11.529199
C	3.108296	-12.200110	-11.530632
C	5.587067	34.979686	-11.531437
C	-1.845982	-10.769788	-11.532187
C	17.966539	13.530604	-11.532541
C	9.300840	-14.352225	-11.533139
C	8.061754	-12.203488	-11.533958
C	4.347152	19.966498	-11.534330
C	16.728387	15.676930	-11.535776
C	15.491160	17.823440	-11.536338
C	21.683215	8.525695	-11.537264
C	4.347601	32.835127	-11.537309
C	-0.601170	11.383286	-11.538327
C	11.775413	-12.921506	-11.538747
C	9.298481	-12.919541	-11.539049
C	14.254072	19.969899	-11.539206
C	-3.083009	-10.056373	-11.540262
C	3.108699	30.690359	-11.540359
C	-0.606204	15.675247	-11.540756
C	-1.845483	17.820450	-11.540853
C	6.821671	-12.917298	-11.540991
C	19.207439	-1.485068	-11.541059
C	8.063591	-16.498860	-11.541696
C	8.062699	22.112231	-11.542638
C	11.776507	21.400123	-11.542813
C	1.868379	29.976537	-11.544480
C	0.631552	-12.200711	-11.544570
C	-3.071578	7.088650	-11.544757
C	5.585606	22.111626	-11.544836
C	-4.324015	19.252234	-11.545085
C	-3.084061	-8.623514	-11.545894
C	10.538655	-12.205992	-11.546934
C	4.345545	21.397786	-11.546984
C	4.344928	-12.915641	-11.547568
C	8.060881	-15.066017	-11.548002
C	15.490838	19.256271	-11.548491
C	14.253489	21.402425	-11.548564
C	16.728497	-0.054153	-11.548617

C	8.063178	-7.907891	-11.550361
C	4.348271	-22.935935	-11.550392
C	20.443242	10.670935	-11.552170
C	-1.845335	-12.202654	-11.552293
C	9.300340	-10.057332	-11.553095
C	10.539614	22.113451	-11.553459
C	3.109353	17.821750	-11.554313
C	3.107286	19.252863	-11.554532
C	11.773913	4.236258	-11.554695
C	6.826143	-18.645060	-11.554945
C	-0.608412	17.106729	-11.556997
C	13.015266	-12.207938	-11.557458
C	13.016341	22.115569	-11.558096
C	16.727868	17.109518	-11.558775
C	1.868293	-12.915411	-11.559516
C	15.491403	0.659597	-11.559774
C	-10.510616	2.804074	-11.560328
C	19.203913	12.816736	-11.560340
C	6.823826	-14.350222	-11.560800
C	5.587665	-20.790731	-11.561591
C	5.584068	23.542774	-11.562511
C	6.824449	34.266255	-11.562730
C	9.300169	7.096682	-11.562894
C	17.965509	14.962913	-11.562936
C	1.869861	28.545504	-11.563197
C	-0.608398	-12.916624	-11.564420
C	3.108480	22.111111	-11.564657
C	-1.847361	19.251902	-11.564885
C	3.107021	27.831993	-11.565493
C	4.345683	29.977077	-11.566266
C	4.346962	24.256135	-11.566841
C	6.823464	-17.212572	-11.567368
C	10.536780	-10.773654	-11.567923
C	-3.084296	19.965961	-11.568851
C	3.106917	23.542306	-11.568944
C	-1.845918	-6.474145	-11.569164
C	3.108722	14.963756	-11.569240
C	-0.606058	-5.757034	-11.570508
C	3.108448	26.400838	-11.570990
C	4.346441	-21.504149	-11.571001
C	4.345475	25.687442	-11.572632
C	1.869964	19.966156	-11.573679
C	4.347063	28.545829	-11.573899

C	0.630827	14.962593	-11.574056
C	4.347576	12.819347	-11.574484
C	5.584520	32.121901	-11.575352
C	1.868325	21.397285	-11.575495
C	8.061213	23.543488	-11.576560
C	5.585352	-19.358670	-11.578856
C	9.299006	-8.624942	-11.579055
C	1.869394	17.107990	-11.579328
C	0.631399	17.821124	-11.579397
C	6.824154	24.256765	-11.579921
C	0.629807	19.252510	-11.579999
C	0.633024	13.530834	-11.581193
C	-0.607339	19.965869	-11.581326
C	1.868309	25.687014	-11.581713
C	-0.608010	-14.349667	-11.582176
C	1.869788	24.255848	-11.582561
C	5.583656	-15.064337	-11.582649
C	4.346584	-14.348703	-11.582781
C	3.108152	-20.790247	-11.583330
C	17.967759	-2.198370	-11.584322
C	-4.321132	-7.911247	-11.585008
C	13.015313	23.547502	-11.585523
C	1.871099	15.676809	-11.585922
C	6.826011	-5.758496	-11.589232
C	0.631256	22.110813	-11.590296
C	5.585435	26.401309	-11.590358
C	0.629956	27.831928	-11.590396
C	5.585689	30.690874	-11.591556
C	10.538320	23.544785	-11.592549
C	5.585924	-16.497225	-11.593246
C	1.869256	-14.348399	-11.594020
C	-0.608844	21.397182	-11.594298
C	13.013426	-10.776320	-11.595909
C	0.629758	23.542172	-11.597122
C	6.824989	32.835512	-11.597813
C	5.583923	27.832480	-11.598611
C	0.628922	-15.063904	-11.599220
C	0.629631	-16.497032	-11.600405
C	1.868317	-18.643929	-11.600697
C	0.631481	26.400645	-11.601088
C	3.111034	13.533172	-11.601093
C	-3.085929	21.397451	-11.601955
C	3.106428	-19.358125	-11.602660

C	11.776818	-10.060940	-11.603137
C	3.106433	-15.063439	-11.603144
C	6.822521	25.687885	-11.603346
C	16.730899	-1.484471	-11.604302
C	4.347432	-18.644102	-11.605022
C	9.301007	24.257718	-11.605171
C	8.062049	-6.475819	-11.605240
C	4.345443	-17.211358	-11.610324
C	10.538964	4.952186	-11.610431
C	5.586687	10.675136	-11.610440
C	13.013823	2.092206	-11.611242
C	-1.845876	22.111001	-11.611997
C	1.867083	-17.211328	-11.612743
C	11.778100	24.259885	-11.613497
C	17.969591	-3.629299	-11.614895
C	3.107898	-16.496462	-11.616761
C	-5.553947	4.233247	-11.620995
C	14.253622	-10.063197	-11.621325
C	-8.032419	2.804011	-11.621349
C	-9.274013	2.091209	-11.621351
C	4.347622	-4.322813	-11.623827
C	-0.606951	24.255828	-11.624266
C	1.869920	-4.322733	-11.628564
C	1.870559	12.819494	-11.630931
C	-0.608232	25.687107	-11.633105
C	-4.322464	-6.479822	-11.635987
C	-4.312419	4.946575	-11.637458
C	-1.847146	23.542217	-11.637733
C	6.823596	28.546365	-11.638419
C	6.822199	29.977577	-11.639442
C	-3.082755	-5.763334	-11.641923
C	8.062091	26.401748	-11.643071
C	10.538997	-7.913357	-11.644463
C	0.633851	10.672523	-11.646731
C	4.350552	11.389559	-11.647472
C	9.299220	25.688604	-11.647869
C	11.775356	-8.629428	-11.649872
C	-0.600737	8.525463	-11.651009
C	11.778237	2.807697	-11.657591
C	14.253724	-0.052073	-11.659883
C	8.061099	32.122579	-11.659990
C	11.776512	25.690850	-11.666273
C	14.252399	-8.632118	-11.667002

C	16.730388	-4.342603	-11.671651
C	8.060162	27.832913	-11.672432
C	15.492651	-7.918853	-11.674618
C	16.731722	-5.773952	-11.675620
C	6.826914	8.531869	-11.677270
C	-0.607040	-4.327458	-11.680718
C	1.872559	11.388230	-11.683199
C	15.492570	-2.197001	-11.684801
C	8.061595	30.691465	-11.686927
C	6.824753	-4.328490	-11.688100
C	-9.274922	0.661421	-11.688617
C	3.110548	-3.609652	-11.690355
C	10.538899	26.402809	-11.690360
C	-5.559902	-5.768894	-11.691534
C	13.015764	-7.917487	-11.692854
C	9.301550	-5.765828	-11.697053
C	13.017874	0.663135	-11.700850
C	15.491934	-6.487510	-11.707414
C	10.537715	-6.482529	-11.707442
C	-1.838920	6.380499	-11.709889
C	0.636676	9.242072	-11.710782
C	5.587923	-3.612543	-11.713328
C	5.590746	9.246817	-11.718218
C	15.494619	-3.628254	-11.719483
C	-6.797378	2.092629	-11.724524
C	0.633197	-3.612515	-11.726039
C	14.256823	-1.482253	-11.726499
C	9.299113	28.546603	-11.732822
C	3.111456	10.677478	-11.734006
C	-3.084140	-4.333775	-11.738383
C	-5.556206	2.806236	-11.742376
C	10.536428	27.833214	-11.748241
C	9.297328	29.977996	-11.749826
C	13.015230	-6.486805	-11.751383
C	8.067864	6.389471	-11.752700
C	-0.599999	7.096781	-11.757336
C	14.256083	-5.773424	-11.757894
C	-5.561734	-4.339190	-11.770545
C	11.778235	-5.771696	-11.773376
C	-1.844052	-3.618456	-11.774864
C	-8.039811	-0.050988	-11.776075
C	14.255575	-4.341618	-11.778693
C	9.300627	-4.335962	-11.782574

C	9.305346	4.244163	-11.786481
C	8.064451	-3.619619	-11.788714
C	-6.799826	-3.627664	-11.801868
C	6.832001	7.104847	-11.805609
C	-3.080308	4.238870	-11.805989
C	-4.321767	-3.624206	-11.808538
C	-8.039830	-1.482839	-11.808541
C	-6.799426	0.663331	-11.810071
C	10.543545	2.098291	-11.810859
C	13.018743	-2.194847	-11.815701
C	3.113382	9.247906	-11.818588
C	11.781420	-0.047986	-11.822041
C	1.872641	8.532940	-11.825808
C	13.019586	-3.626803	-11.826784
C	11.778503	-4.340321	-11.831746
C	4.352881	8.535536	-11.838288
C	-1.840273	4.954111	-11.847023
C	10.541501	-3.625195	-11.850490
C	-6.802419	-2.196559	-11.851801
C	3.109611	-2.184480	-11.856139
C	5.587240	-2.185921	-11.856443
C	8.071512	4.960460	-11.860167
C	11.783158	-1.479541	-11.863616
C	9.308669	2.814582	-11.878230
C	10.546381	0.667844	-11.880321
C	-4.322936	2.096397	-11.882951
C	0.632084	-2.187377	-11.889608
C	8.064299	-2.190382	-11.893064
C	-4.324328	-2.194824	-11.898932
C	10.542502	-2.193169	-11.899957
C	-5.564413	-0.049569	-11.900508
C	-5.562706	-1.482129	-11.902523
C	0.633987	6.389039	-11.909560
C	-1.845963	-2.191536	-11.909660
C	1.875019	7.103703	-11.917507
C	4.350550	-1.473160	-11.930761
C	-3.082923	2.811354	-11.931684
C	6.828112	-1.475346	-11.932890
C	9.305706	-1.478231	-11.935036
C	4.355387	7.106683	-11.938313
C	5.594462	6.394383	-11.945703
C	-4.324130	0.665757	-11.952180
C	1.872891	-1.474407	-11.956960

C	-3.084540	-1.480766	-11.958958
C	9.307135	-0.045142	-11.964676
C	-0.605632	-1.478002	-11.980057
C	3.113677	6.393582	-11.997839
C	6.834533	4.249806	-12.001697
C	8.071025	2.103275	-12.002088
C	-0.607430	4.246381	-12.014321
C	8.070999	0.669658	-12.017765
C	-3.087537	-0.048913	-12.021259
C	6.828978	-0.045009	-12.025976
C	0.634549	4.960860	-12.028389
C	5.596746	4.964373	-12.037428
C	6.834933	2.817696	-12.053150
C	4.350856	-0.044803	-12.061854
C	-1.849698	2.100680	-12.077051
C	5.592621	0.670006	-12.079299
C	3.115479	4.963644	-12.091006
C	-1.847545	0.666196	-12.095252
C	1.872354	-0.047416	-12.105605
C	-0.608302	-0.049710	-12.109362
C	5.593477	2.102834	-12.114924
C	4.354911	4.250769	-12.115054
C	1.872716	4.249666	-12.122942
C	-0.607715	2.816676	-12.124534
C	3.113998	0.668480	-12.134066
C	4.355512	2.817375	-12.152091
C	0.633969	0.665928	-12.169710
C	3.113445	2.102132	-12.182479
C	1.873675	2.817051	-12.189282
C	0.631508	2.102217	-12.199640

References

1. MountainsSPIP, Digital Surf, 25000 Besançon, France. https://www.digitalsurf.com.
2. J. P. Lewis, P. Jelínek, J. Ortega, A. A. Demkov, D. G. Trabada, B. Haycock, H. Wang, G. Adams, J. K. Tomfohr, E. Abad, H. Wang and D. A. Drabold, *Phys. Status Solidi B*, 2011, **248**, 1989-2007.
3. C. Gonzalez, E. Abad, Y. J. Dappe and J. C. Cuevas, *Nanotechnology* **27**, 105201 (2016)
4. Q. Zhang, L. Liu, S. Tao, C. Wang, C. Z. Zhao, C. González, Y. J. Dappe, R. J. Nichols, and L. Yang, *Nano Lett.* **16**, 6534 (2016).
